# Genome-wide analysis of *Homo sapiens*, *Arabidopsis thaliana*, and *Saccharomyces cerevisiae* reveals novel attributes of tail-anchored membrane proteins

**DOI:** 10.1186/s12864-019-6232-x

**Published:** 2019-11-11

**Authors:** Glauber Costa Brito, Wiebke Schormann, Satinder K. Gidda, Robert T. Mullen, David W. Andrews

**Affiliations:** 1 Biological Sciences, Sunnybrook Research Institute, Toronto, ON M4N 3M5 Canada; 2 Department of Molecular and Cellular Biology, University of Guelph, Guelph, ON N1G 2W1 Canada; 3 Departments of Biochemistry and Medical Biophysics, University of Toronto, Toronto, ON Canada

**Keywords:** Tail-anchored membrane proteins, Hydrophobicity scales, RefSeq, Sodium carbonate extraction, BiFC, GO enrichment analysis

## Abstract

**Background:**

Tail-anchored membrane proteins (TAMPs) differ from other integral membrane proteins, because they contain a single transmembrane domain at the extreme carboxyl-terminus and are therefore obliged to target to membranes post-translationally. Although 3–5% of all transmembrane proteins are predicted to be TAMPs only a small number are well characterized**.**

**Results:**

To identify novel putative TAMPs across different species, we used TAMPfinder software to identify 859, 657 and 119 putative TAMPs in human (*Homo sapiens)*, plant (*Arabidopsis thaliana)*, and yeast (*Saccharomyces cerevisiae*), respectively. Bioinformatics analyses of these putative TAMP sequences suggest that the list is highly enriched for authentic TAMPs. To experimentally validate the software predictions several human and plant proteins identified by TAMPfinder that were previously uncharacterized were expressed in cells and visualized at subcellular membranes by fluorescence microscopy and further analyzed by carbonate extraction or by bimolecular fluorescence complementation. With the exception of the pro-apoptotic protein harakiri, which is, peripherally bound to the membrane this subset of novel proteins behave like genuine TAMPs. Comprehensive bioinformatics analysis of the generated TAMP datasets revealed previously unappreciated common and species-specific features such as the unusual size distribution of and the propensity of TAMP proteins to be part of larger complexes. Additionally, novel features of the amino acid sequences that anchor TAMPs to membranes were also revealed.

**Conclusions:**

The findings in this study more than double the number of predicted annotated TAMPs and provide new insights into the common and species-specific features of TAMPs. Furthermore, the list of TAMPs and annotations provide a resource for further investigation.

## Background

Integral membrane proteins contribute in various ways to key cellular functions thus, it is not surprising that 20–30% of the eukaryotic proteome consists of these proteins [1]. Amongst the different types of integral membrane proteins, tail-anchored membrane proteins (TAMPs) differ from other membrane proteins in topology with respect to membranes and the associated cellular constraints on targeting. The defining characteristics of TAMPs are a single transmembrane sequence (TMS) consisting of 15–22 hydrophobic amino acids (aa) located at or near the protein’s carboxyl terminus (C-) terminus, followed by a relatively short (< 30 aa) C-terminal sequence (CTS). In most TAMPs, the targeting information is located in the TMS and CTS [2], although the amino (N-) terminus of the TMS (i.e., NTS) also contains targeting information in some TAMPs [3].

In general, TAMPs are anchored in the membrane by their tail-anchored (TA) sequence with the CTS protruding into the organelle’s interior, and the bulk of the protein, including the functional domain(s) facing the cytoplasm. This topological arrangement is typically important for TAMPs to take part in key cellular processes, like apoptosis [4], vesicular trafficking [3] and protein translocation [5]. To date, approximately 400 proteins of diverse functions have been predicted to be TAMPs [6]. They are found at nearly all organelles, including mitochondria, Golgi apparatus, endoplasmic reticulum (ER), chloroplasts and peroxisomes. In recent years, multiple pathways and targeting machineries have been discovered that mediate TAMP targeting to subcellular locations, in particular to the ER via the TRC40/ASNA1/Get3 pathway [7], and recently discovered alternate SND (SRP-independent) targeting pathway [8] (reviewed in [9]). Although, these targeting machineries have been identified, the signals within the tail sequence that direct TAMPs to different membranes within the cell are not well understood. This complexity of targeting and the repertoire of targeting machineries represent a remarkable level of regulation for a relatively small number of proteins.

One reason that so many open questions remain regarding the spectrum of TAMP functions and the molecular mechanisms involved in their subcellular localization is that the number of identified substrate proteins that have been characterized, is relatively small (< 50). Therefore, identifying additional TAMPs may reveal novel cellular functions that can only be regulated by proteins with this unique topology, may contribute to a better understanding of how TAMP targeting machineries function and/or enable discovery of their evolution. However, to identify putative TAMPs amongst all the proteins encoded by a genome, a bioinformatics approach is required.

Previously, the identification of TAMPs in various genomes, including those from yeast [10], human [6] and plant [11], revealed relatively small numbers of characterized proteins and larger numbers of unknown or predicted proteins. Since that time, some of the predicted proteins have been verified and annotated suggesting that much can be learned by re-examining TAMPs at the whole genome level. As a result, new TAMP prediction tools have been developed. For instance, Shigemitsu et al. [12] formulated a machine-learning based technique to predict TAMPs from tail sequences using Hidden Markov Models [12]. However, this method showed reduced accuracy despite predicting a few TAMPs that were not previously predicted using conventional methods, such as those used by Kalbfleisch et al. [6]. In the latter study, the authors filtered out candidate proteins containing a signal peptide, or those with an N-terminal mitochondrial import signal, and used lists of known TAMPs to derive frequency histograms of amino acids occurring in the TMS to search for a distinctive profile of amino acid distributions. While this approach recapitulated what was already known, little new was revealed beyond a distinctive composition of the TMS in SNARE (soluble N-ethylmaleimide-sensitive factor-activating protein receptor) proteins.

Here, we used a sequence analysis software package called TAMPfinder that can be used to identify putative TAMPs from RefSeq data for *Homo sapiens*, *Arabidopsis thaliana*, and *Saccharomyces cerevisiae* or from UniProt data for other organisms. Using small groups of control proteins and the software user interface we implemented a user-defined scheme for identifying putative TAMPs. To validate the results, a subset of predicted *H. sapiens* TAMPs was examined for localization to subcellular membranes by fluorescence microscopy and/or for integration into the bilayer by biochemical analyses with sodium carbonate extraction [13]. In addition, a set of predicted plant TAMPs were shown to have TAMP features when expressed in tobacco Bright Yellow (BY)-2 suspension-cultured cells. Examination of the entire dataset of sequences for bioinformatics evidence indicated successful enrichment of Gene Ontology (GO) terms and GO-based physical interactions consistent with the identification of TAMPs.

To compare the sequences of different putative TAMPs we established arbitrary borders based on the sequence characteristics and used them to align the sequences. Analyses of the resulting dataset suggested that *H. sapiens* TAMPs possess a median CTS nearly twice as long as the median CTS of *A. thaliana* and *S. cerevisiae* TAMPs. Also, we observed that in *H. sapiens* and *A. thaliana* TAMPs tryptophan is overrepresented at both ends of the TMS (NTS and CTS), while tyrosine is overrepresented just amino-terminal of the CTS. Moreover, the two sets of proteins, containing tryptophan or tyrosine are mutually exclusive. Notably, negatively-charged amino acids (i.e., glutamic acid (Glu), aspartic acid (Asp)) are underrepresented across the entire tail region. Finally, one TAMP, harakiri (HRK) has all the characteristics of a mitochondrially-targeted TAMP yet when expressed in cells the protein is only peripherally bound to the membrane suggesting that the tail anchor (TA) sequence of a TAMP may not always adopt a transmembrane topology.

## Results

### Development of the TAMPfinder classifier and in silico validation

To identify TAMPs within different genomes, a classifier was established via the user interface in TAMPfinder software based on positive and negative attributes of the key characteristics of TAMPs. The software uses numerical values assigned to amino acids to search for strings of amino acids with numerical definitions based on the assigned values. Seven different hydrophobicity scales are built in or optionally it is possible to assign custom values to amino acids individually. Sequences are then identified using a top-hat filter of user defined size and two thresholds. The first threshold identifies a sequence of interest. The second threshold is used to estimate the size of the region identified. Sequences that meet both user defined criteria can be used to select or reject proteins (for details see methods).

To identify TAMPs we searched for a protein sequence with the following characteristics: 1) the identification of a hydrophobic sequence (i.e., TMS) near or at the C-terminus of the protein, 2) the lack of a secretory signal peptide, defined as a moderately hydrophobic sequence at the N-terminus of the protein; and 3) the lack of a predicted TMS elsewhere in the protein. Each of the above mentioned features are defined by a metric of hydrophobicity. However, there is no perfect definition of hydrophobicity and there are multiple scales used to assign hydrophobicity to a polypeptide sequence. Consequently, to take advantage of potential nuances in hydrophobicity scales when training a classifier, we compared seven different scales, namely White [14], Kyte-Doolittle [15], Engelman-Steitz [16], Hopp-Woods [17], Eisenberg [18], Janin [19] and Chothia [20], for their ability to discriminate TAMPs from other proteins based on the defining features described above. Thus, to identify a ‘TAMP-like’ hydrophobic sequence near or at the C-terminus a classifier was generated from a training set of 41 well-characterized TAMPs and 124 well known cytoplasmic proteins (Additional file 1: Table S1). For this purpose an intersection set of proteins with hydrophobic sequences identified using the Hopp-Woods and Kyte-Doolittle hydrophobicity scales provided the best results (Table 1).

**Table 1:** Overview of training and tuning set

Training set	Tuning set
41 TAMPs	41 TAMPs
124 cytoplasmic proteins	63 proteins without signal peptide
36 secreted proteins	96 transmembrane proteins
96 type I transmembrane proteins	54 GPI anchor proteins
Total = 297 proteins	Total = 254 proteins

Secreted and type 1 transmembrane proteins were optimally rejected based on the identification of a putative secretory signal sequence at the N-terminus via a classifier trained with the same 124 cytoplasmic proteins, the 41 TAMPs, and 132 proteins with a signal peptide (36 secreted and 96 well-established Type 1 transmembrane protein sequences) and the White hydrophobicity scale. For rejection of proteins with a second potential TMS, the Engelman-Steitz scale yielded the best classifier for identifying the TMS of the 96 type 1 transmembrane proteins compared to the cytoplasmic proteins and TAMPs. Our goal in constructing this classifier was to be inclusive and to identify novel TAMPs for future analysis. Accordingly, thresholds were selected such that false positives were tolerated at the expense of false negatives. For example, we did not exclude proteins with N-terminal import sequences for mitochondria or plastids, as these import sequences are most often removed post-translationally and, therefore, we reasoned that they could be combined with a TA sequence to anchor a protein in an interior membrane of these organelles. The cut-off for hydrophobicity of the TA sequence was also chosen to favor false positive over false negatives. After optimization and determining the best performing hydrophobicity scale for each segment (Additional file 2: Figure S1), the algorithm was applied to a tuning set of 254 proteins, containing the 41 TAMPs that were part of the initial training set and 254 protein sequences not used for training (Table 1). This set enabled final adjustment to optimize rejection of GPI anchor proteins which similar to TAMPs contain a hydrophobic sequence at the carboxyl-terminus.

When the optimized classifier was used to interrogate the NCBI RefSeq database, approximately 1.6% of the non-redundant known full length entries were identified as potential TAMPs. This is in line with the observation that nearly 5% of all eukaryotic integral membrane proteins are TAMPs [10, 21, 22]. To avoid sequence redundancy, only the longest protein sequence version was kept when there were alternative splicing isoforms. Based on the selected parameters, the program generated a dataset of putative TAMPs for *Homo sapiens* (*n* = 859), *Arabidopsis thaliana* (*n* = 657) and *Saccharomyces cerevisiae* (*n* = 119) (Additional file 3: Table S2). To evaluate the performance of our approach, we measured sensitivity and specificity using a test set comprised of known TAMPs, secreted proteins, nuclear proteins and cytoplasmic proteins (Table 2).

**Table 2:** Validation in silico of TAMPfinder - TP, FP, FN, and TN represent true-positive, false-positive, false-negative, and true-negative values, respectively. Sensitivity = TP/(TP + FN); Specificity TN/(TN + FP)

Positive dataset (experimentally validated TAMPs)				
	*H. sapiens*	*S. cerevisiae*	*A. thaliana*	Total
FN	3	7	11	21
TP	31	31	29	91
Sensitivity	0.912	0.816	0.725	0.813
Negative Dataset 1 (secreted proteins)				
	H. sapiens	S. cerevisiae	A. thaliana	Total
TN	27	6	46	79
FP	11,846	392	1761	13,999
Specificity	0.998	0.985	0.975	0.994
Negative Dataset 2 (nuclear proteins)				
	H. sapiens	S. cerevisiae	A. thaliana	Total
TN	12	2	8	22
FP	1512	823	661	2996
Specificity	0.992	0.998	0.988	0.993
Negative Dataset 3 (cytoplasm proteins)				
	H. sapiens	S. cerevisiae	A. thaliana	Total
TN	4	1	0	5
FP	486	305	203	994
Specificity	0.992	0.997	1.0	0.995

Sensitivity analysis was performed using experimentally validated TAMPs downloaded from Shigemitsu et al. [12]. The sensitivity varied between 0.725 (*A. thaliana*) and 0.912 (*H. sapiens*) and globally was 0.813. As for the specificity analysis, we used 3 different datasets: Negative Dataset 1) included information downloaded from SUBA4 database, designed to store secretome data from *A. thaliana* [23], VerSeDa database for *H. sapiens*, and *S. cerevisiae* [24]. Negative Datasets 2) and 3) included annotations downloaded from GO database, whereby we used both nucleus (Negative Dataset 2) and cytoplasm (Negative Dataset 3) proteins as negative datasets. All of these annotations were based on experimentally-validated subcellular localizations. For Dataset 1 (secreted proteins), specificity varied between 0.975 (*A. thaliana*) and 0.998 (*H. sapiens*), whereas, specificity varied between 0.949 (*A. thaliana*) and 0.994 (*S. cerevisiae*) for Dataset 2 (nucleus) and 0.952 (*A. thaliana*) and 0.980 (*S. cerevisiae*), for Dataset 3 (cytoplasm).

As a final test set we used a list of UniProt protein sequences annotated as TAMPs (single span type IV membrane proteins) from human, plant and yeast which were either experimentally validated or computationally predicted based on similarity metrics. When run on the entire genome, TAMPfinder identified almost 90% of the entries for *H. sapiens* (62 out of 68 see Additional file 4: Table S3). In contrast, previous lists include only 57 of 68 of human TAMPs [6]. In addition, TAMPfinder identified 43 of 67 TAMPs in *A. thaliana* and 24 of 31 TAMPs from *Saccharomyces cerevisiae* from the same UniProt database.

### Validation of putative TAMPs in mammalian cells

**Fig. 1:**
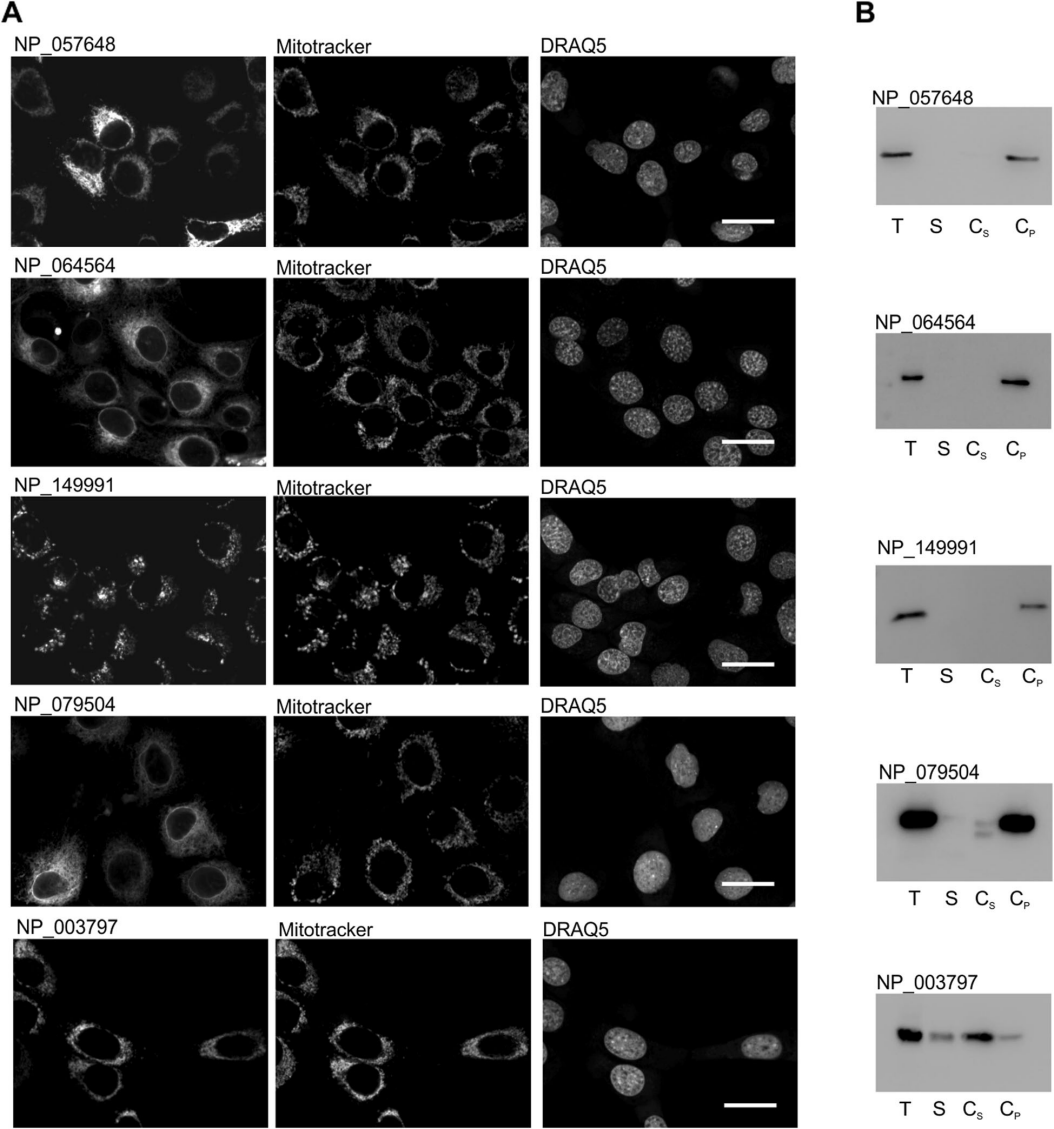
Authentic TAMPs integrate into membrane. **a** Representative confocal microscope images of NMuMG cells expressing the EGFP-tagged putative TA proteins (NP_057648, NP_064564, and NP_149991) and mLumin-tagged NP_079504 and NP_003797. Cells are co-stained with Mitotracker Red (EGFP-NP_057648, EGFP-NP_064564, and EGFP-NP_149991) and Mitotracker Green (mLumin-NP_079504 and mLumin-NP_003797) and nuclear counterstain DRAQ5. Scale bar = 25 μm. **b** By sodium carbonate extraction selected putative TA proteins were tested for membrane integration. Total (T) cell lysate prepared from NMuMG cells stably expressing the indicated EGFP fusion proteins was fractionated into a supernatant fraction (S) containing cytosolic proteins and fractions containing peripheral proteins (C_S_) and sodium carbonate resistant integral membrane proteins (C_P_). Aliquots were analyzed by immunoblotting using antibodies to GFP or RFP (mLumin)

Five putative human TAMPs that lack any published subcellular localization data, and were of different sizes were selected randomly from the TAMPfinder output list as candidates to test for localization at subcellular membranes in live cells and for integration of the putative TA sequence into membranes. The putative TA sequences tested were derived from: glycerol kinase 2 (GK2) (NP_149991), cell cycle exit and neuronal differentiation protein 1 (NP_057648), single-pass membrane and coiled-coil domain-containing protein (NP_064564), TRAF3-interacting JNK-activating modulator isoform 1 (NP_079504), and the pro-apoptotic protein Harakiri (NP_003797). To demonstrate for each of the five proteins that the putative TA sequence was sufficient to mediate localization at subcellular membranes, it was fused to C-terminus of a fluorescent protein (EGFP or mLumin) and the resulting protein was expressed in normal murine mammary gland (NMuMG) cultured cells. When analyzed in this way, the fluorescence protein-tagged-TAMPs showed distinct expression patterns, indicative of localization at specific subcellular membranes. For instances, the TA sequences from GK2, HRK and cell cycle exit and neuronal differentiation protein 1 all co-localized the fluorescence protein with the fluorescent dye Mitotracker, suggesting that these putative TAMPs insert into mitochondrial membranes (Fig. 1a). Sodium carbonate treatment [13] of crude membrane fractions isolated from the NMuMG cells expressing the EGFP/mLumin-TA sequence fusion proteins showed that 4 of the 5 predicted TA sequences are sufficient to anchor EGFP as carbonate resistant integral membrane proteins (Fig. 1b). The exception was HRK, which bound to membranes as it was recovered in the membrane pellet. However when the pelleted membranes were then subjected to carbonate extraction at high pH HRK was released and showed a prominent signal in the sodium carbonate supernatant (Cs) fraction (Fig. 1b). This behavior is considered characteristic of a peripheral membrane protein.

### *A. thaliana* TAMPs target and insert in a TA manner in plant cells

**Fig. 2:**
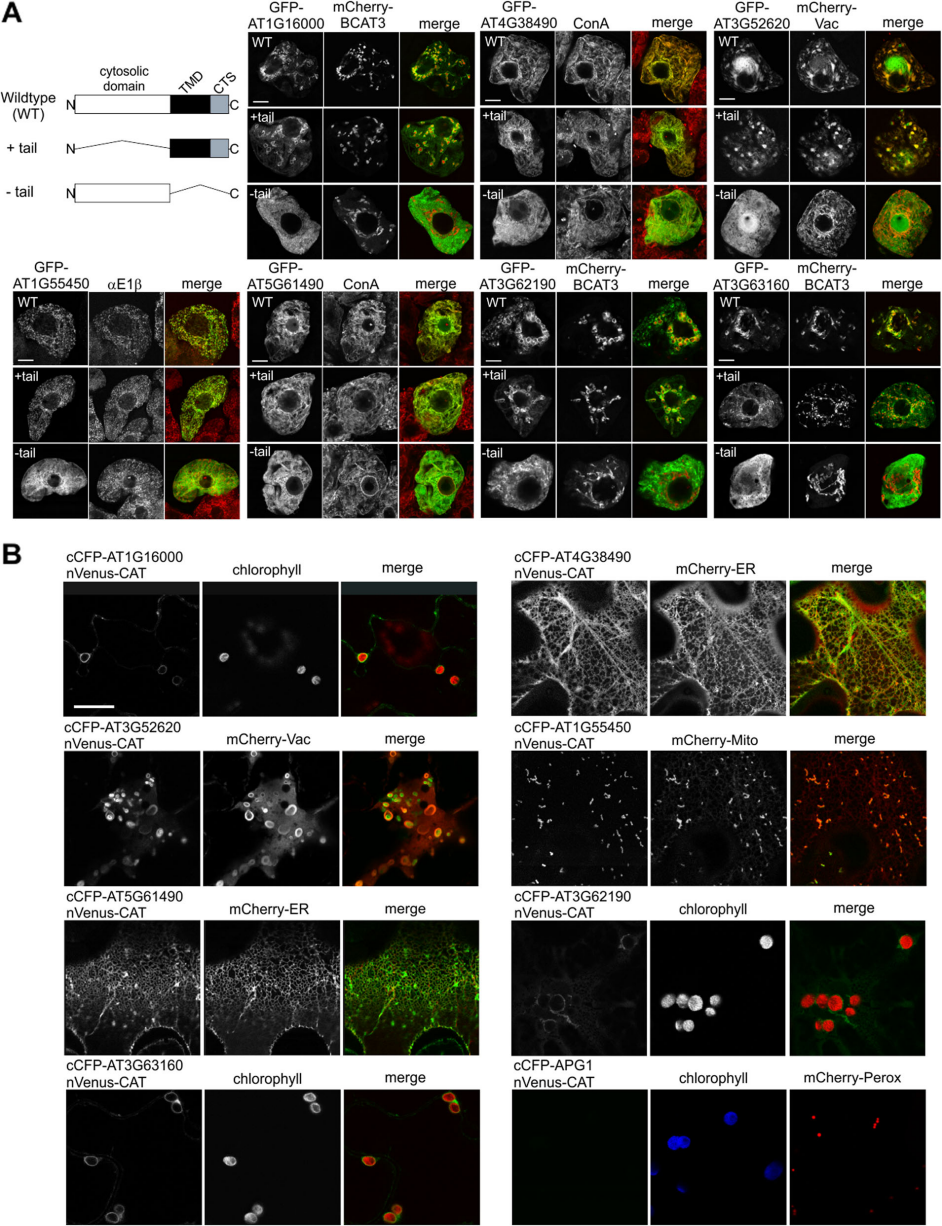
Analysis of subcellular localization and membrane topology of putative plant TAMPs. **a** Targeting analysis by confocal microscopy of plant proteins expressed as GFP fusion proteins in BY2 cells. The constructs expressed are illustrated schematically and included: wildtype, GFP fused to the N-terminus of the complete coding region indicated above the panel; +tail, GFP fused to the ‘tail’ sequence only, i.e., TMD and CTS; and –tail, GFP fused to the rest of the coding sequence without the ‘tail’ sequence. As localization markers the indicated protein (mCherry-BCAT3, mCherry-Vac) was co-expressed or the cells were stained with mCherry-conjugated ConA, or immunofluorescence stained with antibody recognizing E1β. Scale bar = 10 μm. **b** Topology assessment of plant TAMPs in BY2 cells by BiFC. Representative confocal microscope images of plant cells co-expressing cCFP fused to the amino-terminus of the indicated putative TAMP, with the cytoplasmic fusion protein, nVenus-CAT. Shown also are the corresponding images of the chlorophyll autofluorescence, ER marker, vacuole marker, and peroxisome targeting signal (Perox) fused to mCherry. APG1 functions as a negative control. Scale bar = 20 μm

In addition to analyzing novel putative TAMPs in mammalian cells, we extended our validation and studied seven candidates predicted by TAMPfinder to be TAMPs in plant cells (Fig. 2). Three of the *A. thaliana* proteins (AT1G16000, AT1G55450, and AT3G63160) originally identified using TAMPfinder and already characterized thoroughly by us in other studies serve as positive controls in this study. The other four proteins (i.e., AT3G52620, AT3G62190, AT4G38490 and AT5G61490) have not been previously characterized and, with the exception of AT3G62190, which is considered a member of the chaperone DNAJ-domain superfamily, are all annotated at The *Arabidopsis* Information Resource to be transmembrane proteins with unknown function(s). As shown in Fig. 2a, full-length versions of each of the seven *A. thaliana* TAMPs were appended to GFP in a C-terminal manner and expressed in tobacco Bright Yellow (BY)-2 suspension-cultured cells, a well-characterized plant cell system to study protein localization and targeting in vivo [25] and then to verify subcellular localization they were imaged using confocal microscopy. Localization controls included mCherry-fused organelle marker proteins (i.e., BCAT3-mCherry and mCherry-Vac, encoding the plastid stromal branched chain amino transferase 3 and gamma tonoplast intrinsic protein, respectively), immunostaining with primary antibodies raised against mitochondrial matrix E1α along with the corresponding fluorescent dye-conjugated secondary antibodies, or staining with fluorescent dye-conjugated Concanavalin (ConA), an ER-specific stain.

Based on other well characterized tail-anchored proteins [9, 26, 27] and the *H. sapiens* TAMPs analyzed above, the targeting information for plant TAMPs is also expected to be encoded in the C-terminal TA sequence and, consequently, serves as the determinate of the protein’s subcellular localization. To demonstrate that the TA sequence is sufficient for correct targeting, we generated modified GFP fusion proteins of each the seven plant proteins which consisted of only the TA sequence, i.e., the NTS, TMD and CTS appended to GFP (+tail). As shown in Fig. 2a, the localization of each mutant is comparable with that of the corresponding wild-type GFP fusion protein. In contrast, mutants of GFP fusion proteins lacking the C-terminal TA sequence (−tail) were mislocalized to the cytoplasm (Fig. 2a). Taken together, these data indicate that C-terminal TA sequences of the *A. thaliana* proteins examined, including the four newly-identified putative TAMPs (i.e., AT3G62190, AT1G38490, AT5G61490, and AT3G52620) are both sufficient and necessary for their proper targeting in plant cells.

In addition to the subcellular localization and targeting experiments (Fig. 2a), we assessed the membrane topology of each protein, by carrying out a biomolecular fluorescence complementation (BiFC)-based assay that has previously been used to determine membrane protein topology in plant cells [25], To this end, all seven plant proteins were fused at the N terminus to the C-terminal half of CFP (cCFP), whereas the bacterial chloramphenicol acyltransferase (CAT), serving as soluble cytoplasmic marker protein in plant cells [28], was fused to the N-terminal half of the yellow fluorescent protein Venus (nVenus-CAT). The resulting fusion proteins were then transiently co-expressed in tobacco leaf epidermal cells and imaged. The plastid inner membrane protein APG1 (albino or pale green 1) served as a negative control (cCFP-APG1), since its N-terminus (and appended cCFP) is orientated towards the plastid inter-membrane space and therefore inaccessible to the nVenus-CAT protein [29, 30]. AT1G16000, AT1G55450, and AT3G63160, which have been previously characterized in terms of their TA topology [29, 31, 32], served as positive controls in these experiments. To highlight the subcellular localization of reconstituted cCFP:nVenus fusion proteins, cells were also co-transformed with mCherry fused to marker proteins for either the ER (mCherry-ER), mitochondria (mCherry-Mito), or vacuoles (mCherry-γTIP), or to visualize plastids, chlorophyll autofluorescence was imaged. As shown in Fig. 2b, all seven cCFP-appended plant TAMPs co-expressed with nVenus-CAT in tobacco leaf cells displayed a distinct BiFC fluorescence pattern, which also co-localized with their corresponding organelle marker protein, similar to the wild-type GFP fusion protein counterparts (Fig. 2a). By contrast, no BiFC fluorescence was observed in cells co-expressing cCFP-APG1 and nVenus-Cat, as expected. Collectively, these results indicate that all seven *A. thaliana* candidate TAMPs were oriented in a TA manner at the organelle at which they were localized.

### Putative TAMPs are enriched in GO terms associated with membranes

**Fig. 3:**
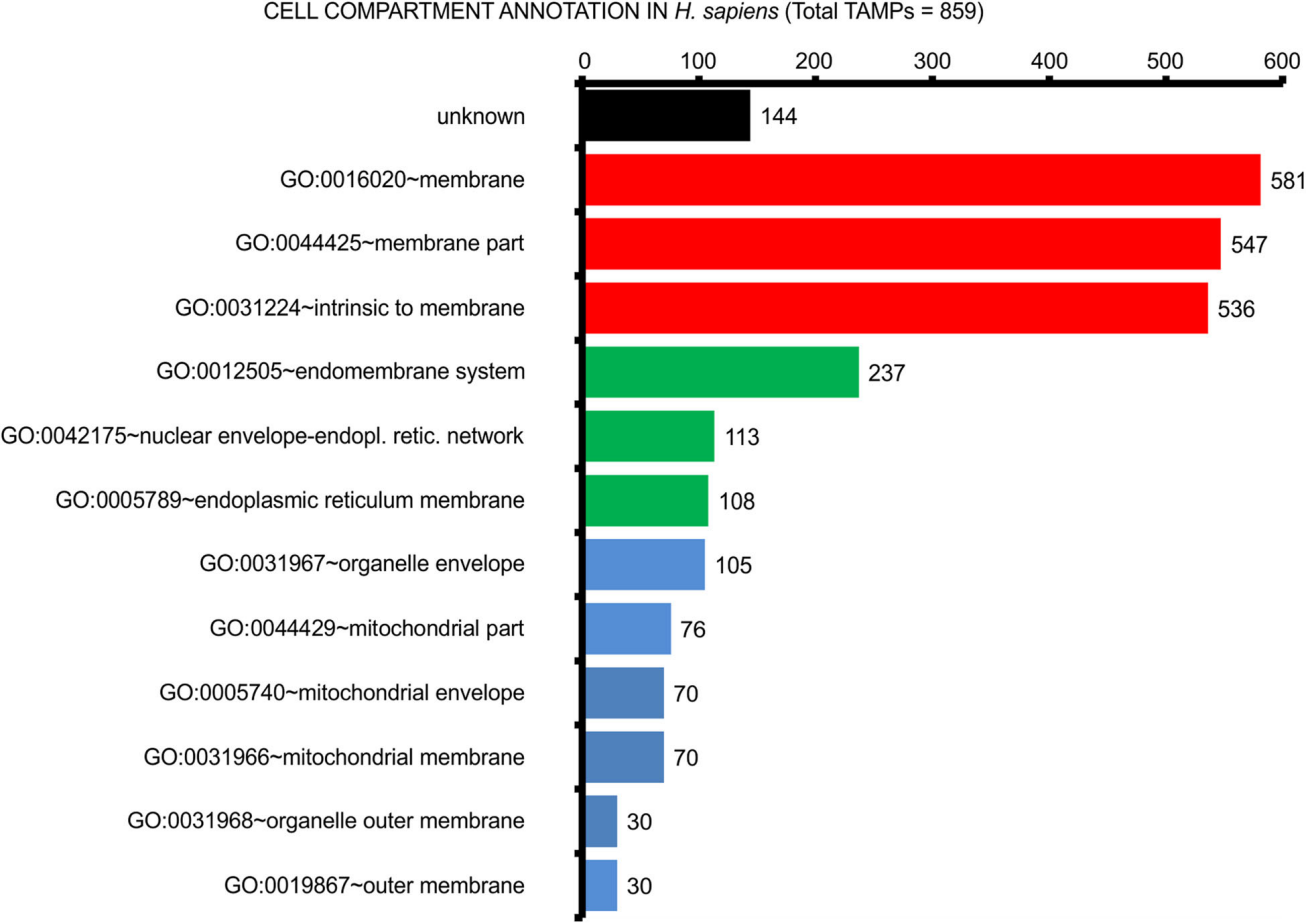
Putative human TAMPs are enriched in GO terms associated with membranes. Only significantly enriched compartments (FDR (False Discovery Rate) < 0.001) are shown. Colors indicate GO terms with similar protein membership. The number of predicted TAMPs with each annotation is indicated to the right of the bars and by the scale above

To further characterize the proteins identified as putative TAMPs, we looked for a statistically significant enrichment in GO terms that are related to “Cellular Component” and “Biological Process” annotations. Compared to earlier methods, TAMPfinder has a global higher coverage of annotated putative TAMPs in *H. sapiens*. For example, while Kalbfleisch et al. [6] found 65% (266/411) of their predicted *H. sapiens* proteins with a GO annotation, 83% (715/859) of TAMPfinder predicted sequences are annotated. The result of the annotated sequences showed that the highest enrichment was for the cell compartment GO terms “membrane” (*n* = 581), “membrane part” (*n* = 547) and “intrinsic to membrane” (*n* = 536) (Fig. 3, red bars). As anticipated, GO terms associated with key organelles, like mitochondria and ER, were also amongst the statistically significant enriched GO annotations (Fig. 3, green and blue). Also, “Cellular Component” GO terms that were highly underrepresented (corrected *p*-value < 0.001) included: nucleus (GO:0005634), nucleoplasm (GO:0005654) and cytoplasm (GO:0005737). TAMPs are also statistically significantly underrepresented in Biological Process GO terms related to binding, metabolism, and regulation of metabolism. Despite TAMPfinder being biased towards false discovery and it identifying more than twice as many putative *H. sapiens* TAMPs as previously identified by Kalbfleisch et al. 2007 (859 vs. 411), the distribution of enriched GO terms for putative *H. sapiens* TAMPs was similar in both screens (Additional file 5: Figure S2). This suggests that the quality of protein predictions (i.e., likelihood that a prediction is correct) was similar. As expected, cell compartment GO terms highly enriched in *H. sapiens* TAMPs (Fig. 3) are also enriched in the *A. thaliana* dataset (Additional file 6: Figure S3). Additionally, functional GO terms related to the characteristic plant organelle (plastid) and its associated molecular processes (photosynthesis) are also significantly overrepresented consistent with reports showing that specific TAMPs localize to this organelle [29]. The underrepresented GO terms (corrected *p*-value < 0.05) in *A. thaliana* include mainly “Metabolic Processes”, such as: cellular metabolic process (GO:0044237), primary metabolic process (GO:0044238), macromolecule metabolic process (GO:0043170), cellular macromolecule metabolic process (GO:0044260), and organic substance metabolic process (GO:0071704).

### Identification of sub-regions in TA sequences

**Fig. 4:**
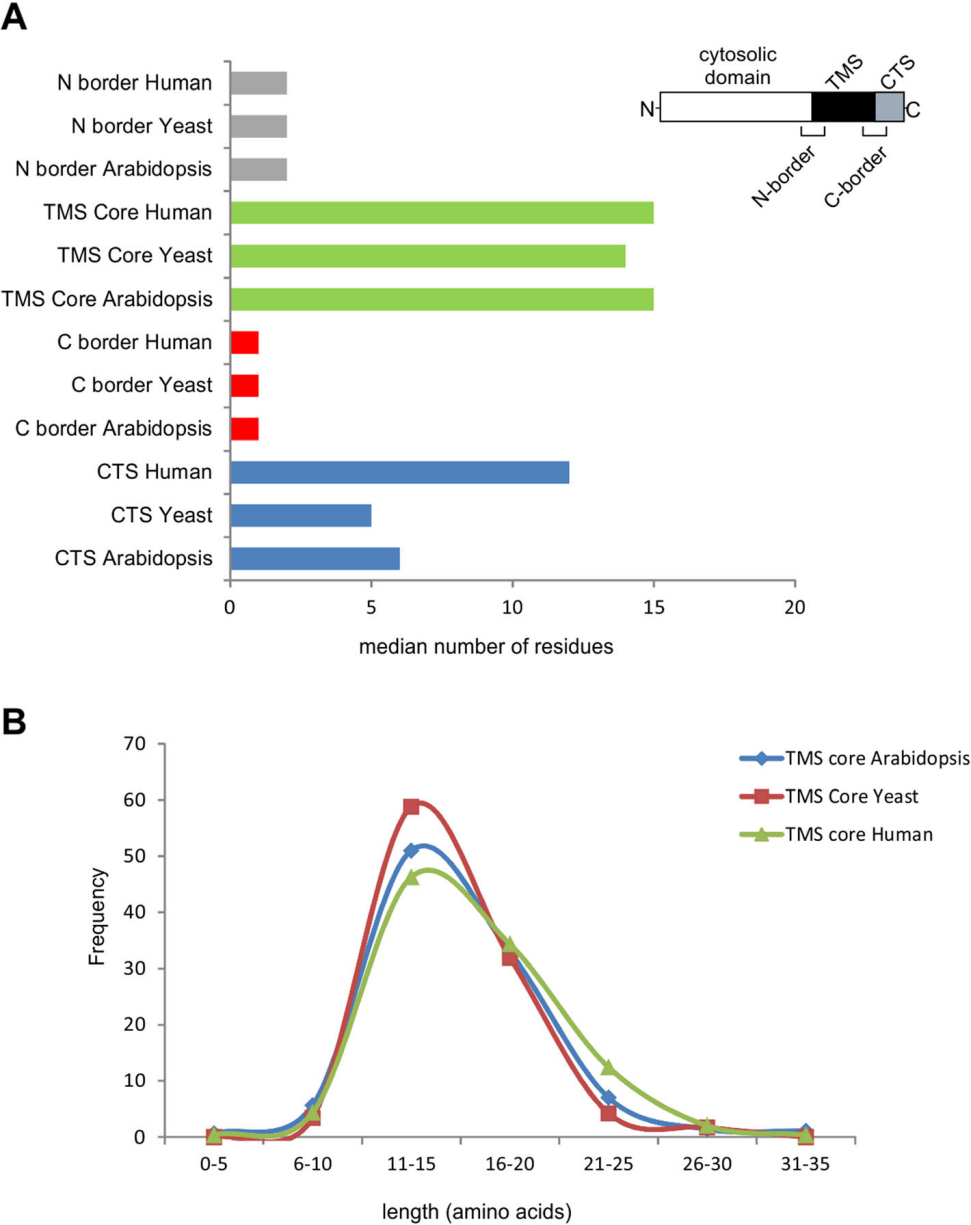
Comparison of the length distributions for sub-regions for TA sequences amongst different species datasets. **a** Number of residues in each region of the tail anchor and **b** frequency of TMS core length in *H. sapiens*, *S. cerevisiae*, and *A. thaliana*, as indicated

A feature of the TAMPfinder program is that it provides numerical definitions for sub-regions of the TA sequence for each putative TAMP (Fig. 4a-b). This permits multiple options for aligning the putative TA sequences. Thus, the hydrophobic residues identified as the intersection of the hydrophobic sequences identified by Hopp-Woods and Kyte-Doolittle hydrophobicity scales was defined as the TMS core. While the hydrophobic sequence identified by each scale was usually similar, it was often not identical and therefore the amino acids at each end of the hydrophobic core identified by only one of the two scales were termed the N and C borders, respectively. If there was no disagreement, then the first amino acid outside of the TMS core was assigned as the border. This approach was extended to provide a numerical definition of NTS as the 15 amino acids N-terminal of the N-border and CTS as comprised of all of the residues carboxyl of the C-border. Plotting the median length for TMS core for *H. sapiens*, *A. thaliana* and *S. cerevisiae* (Fig. 4a) revealed that it consisted of 14–15 residues as expected based on previous definitions of TAMPs [33]. The distribution of hydrophobic TMS core lengths in all three species peaked at 11–15 aa, however in *H. sapiens* the distribution of TMS core lengths was skewed to slightly longer lengths (Fig. 4b). The median length of the border regions was generally, two or less residues for all three species. However for a few proteins the region of disagreement was larger. Thus, the largest between species difference in lengths for the various TA regions was for the CTS. The median CTS for *H. sapiens* (12 residues) was twice as long as the median CTS of *A. thaliana* and *S. cerevisiae* (5–6 residues).

### Distribution of amino acids in the TA region in *H. sapiens* and *A. thaliana*

**Fig. 5:**
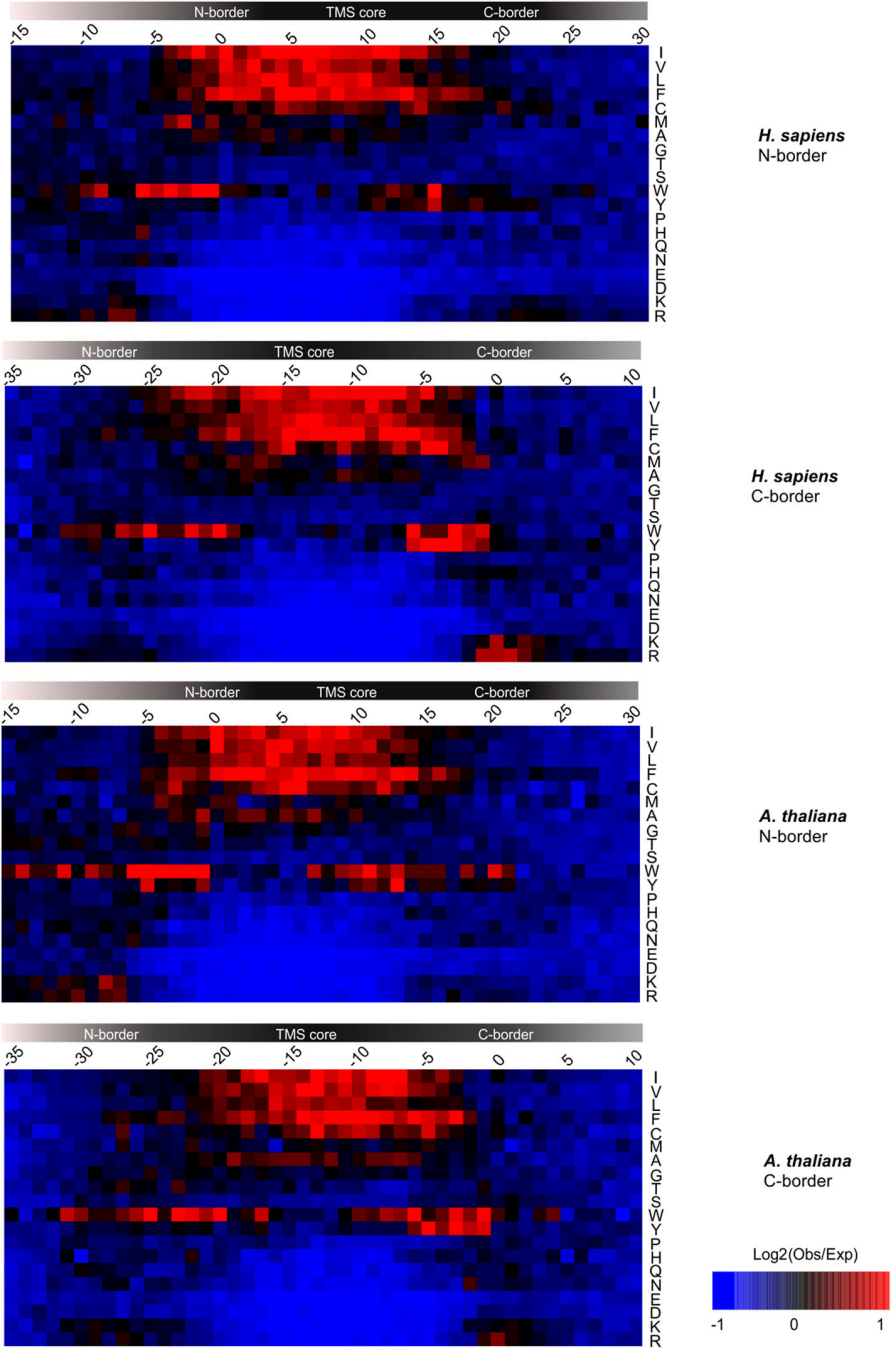
Enrichment of amino acid residues at different positions within the TA sequences. To examine the enrichment of amino acids throughout the TA region, the sequences of just the TA regions were aligned on either the N- or C-border as indicated at the right and the frequency of occurrence was measured for all 20 amino acids at the indicated locations of locations. These frequencies were then compared to the frequency with which the same amino acid was found in the regions outside the TA region, The underrepresentation of amino acids at the amino-ends (first 5 residues) is a result of examining only the small number of TA sequences for which differences in the hydrophobicity scales resulted in an extended N-region (NTS and N-border) and excluding the rest of the protein sequence. A similar phenomenon occurs at the C-terminus due to differences in the numbers of residues in the CTSs. Red indicates overrepresentation; blue indicates underrepresentation according to the scale provided. Amino acid identities are indicated in single letter code at the right of the panels

To examine the distribution of amino acids throughout the TA region, it was necessary to first align all of the TA regions. The high variability in the length of the CTS meant that aligning on the C-terminus of each protein was not appropriate. The center of the TMS has more uncertainty than either end because it is affected by the variability in both of the N- and C-borders. Therefore, the sequences were aligned on either the N- or C-border as these regions had the least variability and the tightest distribution of lengths (Fig. 5). The frequency of occurrence was measured for all 20 amino acids at every position across the TA region. The residue positions examined for sequences aligned on the N-border encompassed a region − 15 to + 30 of the alignment position while for alignment on the C-border locations positions − 35 to + 10 were examined. To identify over- and underrepresented amino acids at each position the frequency of occurrence was compared to the frequency with which the same amino acid was found in the region outside the TA region for all of the selected proteins. The control frequencies were calculated in this way to correct for the possibility that there may be a skewed amino acid prevalence in this subset of proteins. However, for the large sets of proteins examined here, this value was the same as the frequency of occurrence of the amino acid in the entire genome of the species. As expected, for both *H. sapiens* and *A. thaliana* there was a clear overrepresentation of hydrophobic amino acids isoleucine (Ile), valine (Val), leucine (Leu), phenylalanine (Phe) and underrepresentation of charged or polar amino acids glutamic acid (Glu), asparagine (Asn), aspartic acid (Asp), lysine (Lys), arginine (Arg) within the TMS. This representation further suggests that most hydrophobic residues are evenly distributed within the TMS. However, when aligned on the N-border it appears that in both *H. sapiens* and *A. thaliana* the distribution of cysteines (Cys) is uneven with increased representation at the ends and in the middle of the TMS. While in *H. sapiens* methionine (Met) is overrepresented at the N-end of the TMS and underrepresented in the center, in A. thaliana the residue is not enriched throughout. Consistent with other studies on amino acid distributions in membrane proteins [33], tryptophan (Trp) and tyrosine (Tyr), both amino acids with aromatic side chains, are overrepresented. However, unlike other transmembrane proteins in which Trp tends to be located near the cytoplasmic end of transmembrane sequences, in TAMPs Trp is overrepresented at both ends of the TMS while Tyr is only overrepresented just N-terminal of the C-border. When compared it seems that Trp tends to be more enriched in *A. thaliana* than in *H. sapiens* at specific positions for both alignments (Additional file 7: Figure S4). To determine if there was a relationship between the distributions of Trp and Tyr residues we aligned all of the sequences with a Trp near the N-border on either the N-border or the Trp residue and performed the analysis again on this subset of the proteins. While the overall distribution of amino acids was similar in these alignments of proteins with a Trp near the N-border the enrichment in Tyr disappeared. The enrichment for Tyr was also lost for sequences containing Trp near the C-border when aligned on the C-border or the Trp near the C-border. Finally when the sequences containing a Tyr near the C-border were aligned on the Tyr or the C-border there was no longer enrichment in Trp anywhere in the sequence. Thus, we conclude that the Trp and Tyr are mutually exclusive (Fig. 5). As seen in Fig. 5, the enrichment in Trp and Tyr extends across several adjacent residues. This may be uncertainty due to the arbitrary alignment method used or it may suggest biological significance. Nevertheless, that Trp is enriched at positions − 1 through − 5 further emphasizes the importance of the observation. As a more rigorous test of the significance of all these observations we determined the statistical significance at each position independently (Additional file 8: Figure S5).

As expected, positively-charged amino acids (Lys, Arg) are overrepresented at the N-end of the TMS (cytoplasmic side of the membrane) in both *H*. *sapiens* and *A. thaliana* (Fig. 5)*.* However, unlike conventional TMSs where positive charges are distributed according to the positive-inside rule [34], in TAMPs, both Lys and Arg were enriched at the C-terminus on the lumenal side of the membrane also. Indeed when aligned on the C-border it is clear that in humans these residues are unexpectedly present at a higher frequency at the C-terminus than at the N-terminus of the TMS core. Even when Lys and Arg are assessed for frequency at the N-terminus when the sequence is aligned on the N-border the enrichment is less than near the C-terminus of the TMS (Fig. 5). Another unexpected result was underrepresentation of negatively-charged amino acids (Asp, Glu) in all areas of the TA sequence in both *H*. *sapiens* and *A. thaliana.*

### TAMPs are shorter than other proteins

**Fig. 6:**
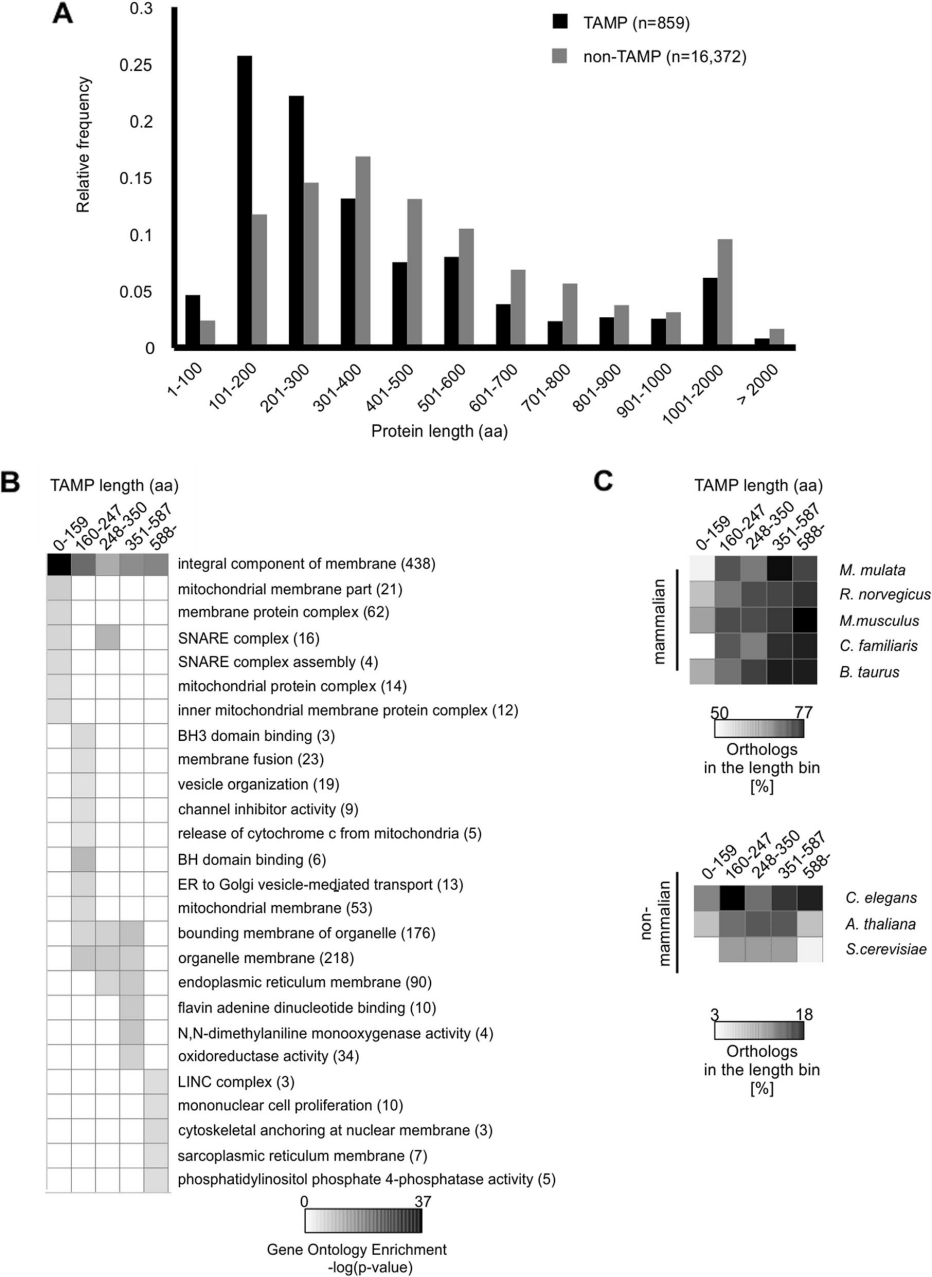
TAMP length associated to GO terms and protein conservation in *H. sapiens.* **a** Frequency distribution of TAMPs and non-TAMPs according to the lengths indicated below. Median length of TAMPs is 288 aa, while non-TAMPs have 432 aa (t-test, *p* < 4e^− 11^). There is a bias exemplified by the shape of the distributions to a shorter length for TAMPs compared to non-TAMPs. **b** Enrichment of GO terms according to TAMP length. Proteins were binned according to their length such that each bin contains 143 proteins and a GO Term enrichment analysis relative to the same term for the human genome was performed for each length bin. Numbers in parentheses represent the total number of TAMPs annotated with that GO Term. **c** Profile of conservation across different human TAMP lengths. Each cell shows the percentage of the orthologs (a proxy of conservation) of the specified length (columns) from a given species (rows) that are predicted to be TAMPs. The enrichment of orthologs among TAMPs longer than 160 amino acids in both mammalians and non-mammalians suggests that the longer TAMPs are more conserved across species

A simple comparison of median lengths revealed that TAMP and non-TAMP proteins have median lengths of 288 and 432 amino acids, respectively (Fig. 6a). Global comparison of the length distribution of these proteins further illustrated that the length of TAMPs is much more skewed shorter than for other proteins with half of all TAMPs and less than a third of other proteins less than 300 amino acids long (Fig. 6a). The skewed distribution of lengths (Fig. 6a) suggested that there might be an association between TAMP length and function that would provide more granularity than the general GO terms obtained for all TAMPs (Fig. 3). To examine this, we identified statistically enriched GO terms assigned to TAMPs assigned to protein length groups arbitrarily selected to contain 143 proteins each (Fig. 6b). This method divides the proteins such that the first two groups include all the proteins below the median then the next two groups contain equal numbers of proteins above the median and the last group includes all the larger proteins. The first two groups also encompass the protein lengths proportionally more common for TAMPs than other proteins. As expected, we observed enrichment of the GO term integral component of membranes independent of specific protein lengths. However, this analysis also revealed that TAMPs associated with mitochondrial membranes and SNARE complexes tend to be shorter than other TAMPs. On the other hand, TAMPs with GO terms related to the endoplasmic reticulum, sarcoplasmic reticulum and cytoskeleton tend to be longer than the median. Moreover, the group of shortest TAMPs (< 160 residues) is the least conserved across mammalian species (Fig. 6c) a result consistent with previous reports suggesting longer proteins are more conserved [35]. The conservation/length profile of TAMPs showed significant differences compared to other membrane and cytoplasmic proteins (Fig. 6c and Additional file 9: Figure S6). Thus, human TAMPs showed globally a median of 69.2% of mammalian orthologs in all length windows, while membrane and cytosol proteins showed 87.7 and 88.1% (*P* = 5e^− 9^, *P* = 5e^− 10^, respectively), indicating that human TAMPs tend to be less conserved than other mammalian proteins from these cell compartments. Consistent with this data, when we compared conservation of TAMPs and cytoplasmic proteins between *H. sapiens* and *C. elegans, A. thaliana,* and *S. cerevisiae*, we observed half as many orthologs (10.5 and 22.1%, *P* = 1e^− 6^, respectively) for human TAMPs. No significant difference was found between the number of orthologs for human TAMPs and membrane proteins from these species. These results suggest that the evolutionary difference between human TAMPs and other membrane proteins is restricted to mammalians, while cytoplasmic proteins tend to be more conserved than human TAMPs in all evolutionary contexts analyzed.

### TAMPs involved in specific protein-protein interactions (PPIs)

**Fig. 7:**
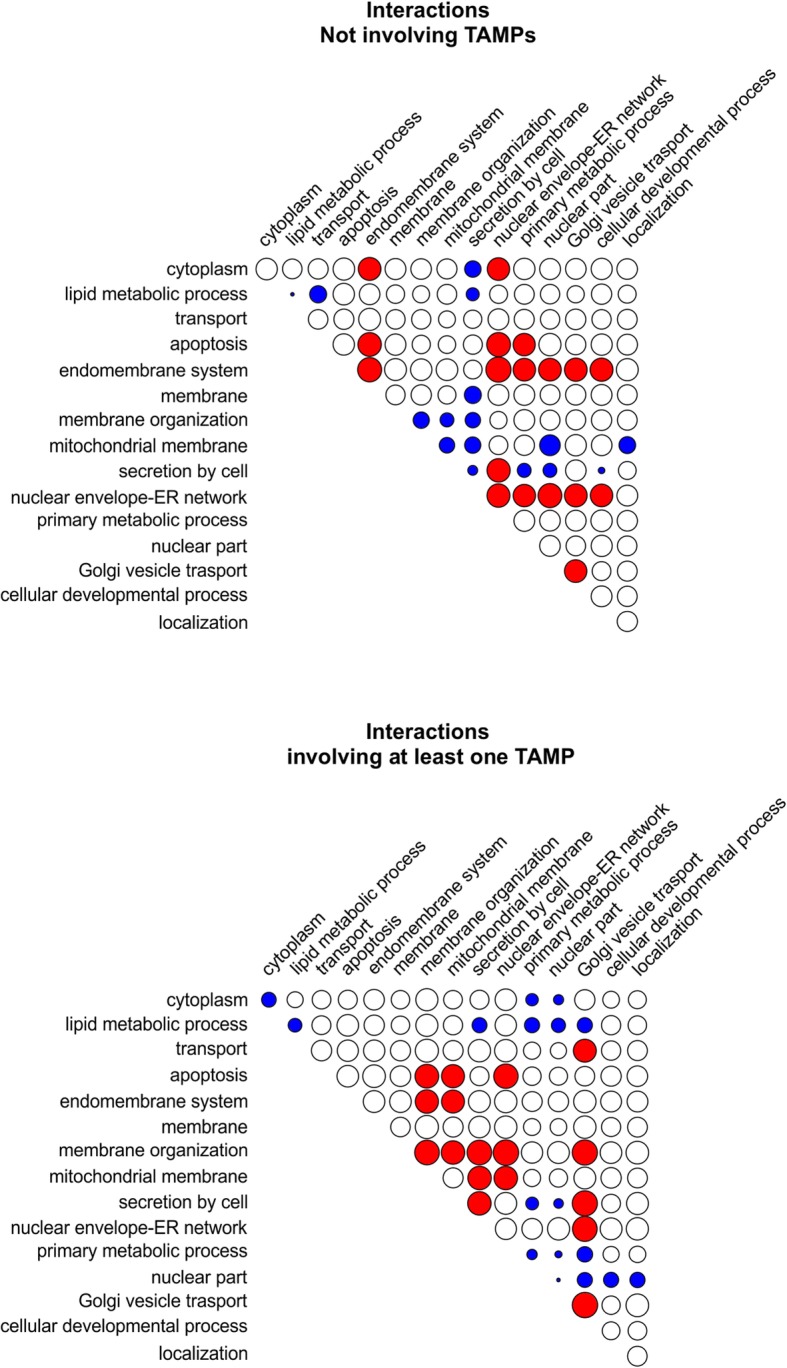
Enrichment of PPIs within and across defined biological processes and cell compartments globally involving TAMPs. The relative number of protein pairs interacting physically was measured for different Gene Ontology categories. A red color indicates interaction above the frequency expected by chance, blue color indicates interaction below the expected frequency and white color, statistically indistinguishable from random interaction. Diagonal shows interaction of proteins from the same Gene Ontology category (symmetric interaction). Enrichment is proportional to the radius of the circles

Examination of TAMP GO terms associated with the TAMPs of specific lengths highlighted the possible involvement of TAMPs in protein complexes (e.g., SNARE complex, mitochondrial protein complex, LINC complex) (Fig. 6c). Taken together with well-studied TAMPs known to be part of the protein translocation complex at the ER, the mitochondrial protein import complex and protein complexes that regulate apoptosis [27] our analysis suggested that TAMPs may be disproportionally involved in protein-protein interactions. In order to analyze this further we examined enrichment in physical protein-protein interactions (PPIs) derived from BioGRID. The interactions were analyzed in terms of enrichment within and across defined biological processes and cell compartments. We classified these interactions into two groups: 1) interactions not involving a TAMP, and 2) interactions involving at least one TAMP. As expected, since TAMPs do not show enrichment of cytoplasm-related GO terms, it was not surprising that the number of interactions in which one of the proteins was assigned the GO term “cytoplasm” was below that expected by chance (Fig. 7). As also expected, BioGRID data analysis revealed enrichment of protein-protein interactions between TAMPs and proteins associated with the GO terms “membrane organization” and “mitochondrial membrane”, compared to the BioGRID dataset that does not include any TAMPs. These results suggest that other interactions (or lack thereof) identified in this way may be biologically relevant. Consistent with apoptotic processes being executed on the mitochondrial membrane [36] and some Bcl-2 family proteins being TAMPs, we found more PPIs involving TAMPs related to apoptosis and proteins associated with the mitochondrial membrane and membrane organization than expected from the non-TAMP dataset. This analysis also suggests that there is also an enrichment of TAMPs in the category “secretion by cell” which may reflect the known role for TAMPs in vesicle budding and transport. Moreover, TAMPs tend not to interact with proteins that are involved either in metabolic processes or nuclear part. At a more global level, we compared the number of PPIs involving only TAMPs with the number of PPIs observed in BioGRID by randomly sampling one million times an equal number of proteins. This analysis revealed an enrichment of interactions involving only TAMPs (*P* < 10e^− 6^) compared to interactions involving other proteins, suggesting that TAMPs are more frequently found in complexes with other TAMPs than other proteins are part of complexes. However, this appears to be largely a function of TAMPs being membrane proteins because the number of protein-protein interactions per TAMP (median 7) is very similar to that of other membrane proteins (median 8). Moreover, there were no significant differences in the number of interacting partners for TAMPs of different lengths.

Many of the enriched and depleted interaction categories are very different for TAMPs compared to other proteins (Fig. 7). For example, although interactions are very common for proteins with the GO term endomembrane system, TAMP interactions with these proteins are restricted to membrane organization and mitochondrial membrane whereas non-TAMP interactions are high in many other categories. Similarly, there is low overlap in enriched GO terms for interactions related to apoptosis. The paucity of interactions between TAMPs and proteins involved in metabolism in general and lipid metabolism specifically suggests that the unique topology of TAMPs is not advantageous for this cellular process (Fig. 7). Perhaps the most striking finding is related to membrane organization where interactions involving a TAMP are enriched for many categories yet depleted for non-TAMPs. This suggests that TAMPs are much more likely than other proteins to be involved in regulating membrane organization.

## Discussion and conclusion

Less than 100 unique human TAMPs have been more than minimally characterized greatly limiting our understanding of this important class of proteins. While based on experiments with this limited set of proteins most TAMPs are believed to target to a single subcellular membrane, Bcl-2 is dual targeted to both the ER [37] and mitochondria [38, 39]. Whether Bcl-2 is truly an exception and how common dual targeting for TAMPs is remain to be determined. Similarly, cytochrome *b*_5_ remains unique in binding to membranes independent of either chaperones and/or identified targeting machinery [40]. Further we demonstrate here that although HRK shares all other features with TAMPs it binds to membranes as a peripheral rather than integral membrane protein. Only by expanding the cadre of TAMPs that are thoroughly examined will the true range of activities and functions of these proteins be discovered. For that reason, we have identified putative TAMPs and provide an annotated list of these proteins. Because little is known about the similarities and differences between the amino acid sequences that function as a TA we included a more precise definition of the sub-regions of the TA sequence. Differences in the sub-regions can be used to identify clusters of related TAMPs as shown for the identification of syntaxin related TAMPs [41]. Here, we report data from *H. sapiens, A. thaliana* and *S. cerevisiae* querying the NCBI RefSeq database (Release 82).

Our list of candidate TAMPs was validated in multiple ways. First we identified 40% (164/411) of the full list of proteins found previously as putative TAMPs by Kalbfleisch et al. [6]. Moreover, the proteins that we did not identify from that list showed 3.5 times fewer predicted membrane proteins (141 vs 499, additional file 10: Table S4), and a much lower enrichment of membrane proteins (*P* = 9e^− 19^ versus *P* = 5e^− 67^) compared to the proteins reported here that were not included in the published list [6]. In addition, our estimated specificity based on independent sequences of secreted, nucleus, and cytoplasmic proteins was 0.981, 0.993, and 0.995, respectively, thus providing confidence that the proteins identified here are highly enriched in TAMPs. Based on experimental verification testing, our list has a sensitivity of 0.813, while the specificity ranged from 0.993–0.995. Finally when used to query the UniProt database the TAMPfinder software identified almost 90% of the TAMP entries for *H. sapiens* many of which were not previously identified (see Additional file 4: Table S3). Hence our approach shows a good performance in discriminating TAMPs from non-TAMPs and is also as effective as the recently published TAMP predictor by Shigemitsu [12], which showed global sensitivity and specificity of 0.864 and 0.952, respectively. However, the authors of the latter study did not provide a precise definition of the N- and C-termini of the TA sequence. Here, we define these sequences as well as the borders between them and the hydrophobic core sequence allowing us to profile the distribution of amino acids across the full sequence. Finally, our list of candidate TAMPs was validated was based on a combination of fluorescence imaging of subcellular localization of EGFP or mLumin attached to selected *H. sapiens* and *A. thaliana* TA sequences (Figs. 1 and 2), and direct membrane binding assays.

In our dataset, 46 and 54% of the *H. sapiens* putative TAMPs are predicted proteins (RefSeq: XP_) or known proteins (RefSeq: NP_) compared to 39 and 61% of other human proteins (RefSeq), respectively (Additional file 5: Figure S2). We processed data from Kalbfleisch et al. [6] and found that 65% (266/411) of their predicted TAMPs showed some sort of GO annotation, while 83% (715/859) of our predicted TAMPs are annotated likely mostly due to changes in the databases in the intervening time period (Additional file 5: Figure S2). As expected, for those TAMPs that are annotated, the enriched GO terms are related to membranes. In contrast, GO terms associated with RNA or DNA related molecular functions are absent for TAMPs. Additionally, TAMPs are not involved in protein-protein interactions with proteins that participate in metabolic processes (Fig. 7). That only a few categories were depleted is consistent with TAMPs being involved in a wide variety of cellular processes.

In contrast to other lists of putative TAMPs [6, 12], we also provide a comprehensive sequence analysis showing both differences and commonalities in TAMPs from different species. A picture of TAMPs emerges from our analysis in which, irrespective of species, and compared to other membrane proteins, TAMPs have several notable features including: i) they are in general shorter, indeed about half are less than 288 amino acids compared to 432 amino acids for other proteins; ii) have a shorter TMS core sequence of 14–15 amino acids instead of the 20–21 typical of transmembrane helices; iii) show enrichment for Trp at either end of the TMS core that is mutually exclusive with enrichment for Tyr near the C-terminus; iv) enriched in Lys and Arg at both ends, instead of primarily at the cytoplasmic end of the TMS core as expected from the positive-inside rule [34]; and v) depleted of Glu and Asp throughout the entire TA region (Fig. 5). The main variation between species was observed in the CTS sequence that was substantially longer for human than for *S. cerevisiae* and *A. thaliana*, 12 versus 5–6, respectively (Fig. 4a).

Although most TAMPs are short, the most highly conserved TAMPs are longer than 160 amino acids. Increased conservation with length is not specific to TAMPs but has been reported for all proteins, irrespective of whether they are annotated as cytoplasmic or membrane proteins (Fig. 6c and Additional file 9: Figure S6) [35]. Nevertheless, the TAMPs with the most specific GO terms are less than 200 amino acids in length including BH3 domain binding, tertiary granule membrane, and SNARE complex assembly etc. This does not mean a priori that specific functions have not been ascribed to any larger TAMPs. For example, the 84 kDa TAMP Golgin84 has been shown involved in generating and maintaining the architecture of the Golgi apparatus [42]. Nevertheless, the data showing that many highly conserved TAMPs are in the 200–300 kDa range suggests that many of the most important functions of this class of proteins are yet to be discovered. The most well annotated function for the TAMPs identified here that distinguishes them from other proteins is their roles in membrane organization (Figs. 3, 6b and 7). Importantly, the list of TAMPs and annotations both of sequence details and functions provided here and a new set based on the same parameters but more recent sequence data available at [43] provides a resource for further investigation of this enigmatic class of proteins.

## Methods

### TAMPfinder algorithm

The TAMPfinder program (CRC Systems, Burlington) [44] used to identify TAMPs scans the last 60 amino acids of a protein sequence to identify a hydrophobic region. The basic algorithm involves calculating hydrophobicity using one of 7 user selectable scales or by a custom scale in which a value is set for each amino acid by the user. The program then calculates local hydrophobicity using a user defined top hat filter. Hydrophobic segments are defined by using two thresholds, the higher of which defines a potential hydrophobic sequence. A second lower threshold is then used to define the length of the hydrophobic sequence (Additional file 2: Figure S1). The thresholds and size of the top hat filter were optimized to differentiate TAMPs from a set of manually curated TAMP and cytoplasmic proteins. Both the Hopp-Woods (Threshold 1:-0.96, Threshold 2: − 0.6, filter size 9–12) and Kyte-Doolittle scales (Threshold 1: 2.0, Threshold 2: 0.8, filter size 7–11) functioned equivalently to identify bona fide hydrophobic regions as a putative transmembrane sequence core (TMS-core) but had different false positives; therefore, an intersection set of the two was used. Optimization of each of the tests with the different hydrophobicity scales is shown in Additional file 2: Figure S1. The intersection set and the second threshold allowed automated assignment of the amino-border (N-border) and carboxyl-border (C-border) sequences. The carboxyl-terminal sequence (CTS) was defined as all of the residues C-terminal of the C-border while the amino-terminal sequence (NTS) was arbitrarily defined as the 15 amino acids amino-terminal of the N-border. The advantage of automated assignment of these borders was to use them to align the sequences. Preliminary experiments demonstrated that other alignments such as to the position of peak hydrophobicity or the C-terminus of the protein were not useful.

A similar approach was used to identify putative secretory signal sequences within 30 amino acids of the amino-terminus of the protein. In this case the training set included the TAMPs (*n* = 41) and a set of protein sequences known to have an amino-terminal signal sequence. The White hydrophobicity scale was optimal for differentiating the two classes of proteins (Threshold 1: 0.3, Threshold 2: 0, filter size 6–9). The final criterion was that the sequence between the 30 amino acids at the N-terminus and the 60- amino acids at the carboxyl-terminal region not contain a putative TMS. To identify putative transmembrane sequences the same approach was used but the Engelman-Steitz hydrophobicity scale proved optimal (Threshold 1: − 2.1, Threshold 2: − 1.7, filter size 7–8). For protein sequences less than 100 amino acids in length this final search was omitted.

The discriminatory powers of TAMPfinder were indicated by sensitivity and specificity values calculated using independent test sets of sequences:

$$ \mathrm{Sensitivity}=\frac{TP}{TP+ FN.} $$

$$ \mathrm{Specificity}=\frac{TN}{TN+ FP} $$

TP, FP, FN, and TN signify true-positive, false-positive, false-negative, and true-negative values, respectively. These terms are independent of the genome (population of interest) subjected to the test. Therefore, an algorithm with 100% sensitivity correctly identifies all TAMP proteins in the genome, while an algorithm with 100% specificity correctly identifies all non-TAMP proteins. An algorithm with a high sensitivity but low specificity results in many predicted TAMP proteins that are actually non-TAMP proteins. Conversely, low sensitivity but high specificity results in many truly TAMP proteins that are missed.

A web version of the output files containing lists of the TAMPs and their assigned features and software manual are available at [43].

### Amino acid sequence alignment

To compare the sequences of different putative TAMPs requires that we align the amino acid sequences. Since there is no defined way to align these sequences we established arbitrary borders based on the sequence characteristics of the tail. Consequently, the hydrophobic core sequence was designated as TMS based on its proposed topography with respect to the membrane. Sequences located C-terminal of the hydrophobic core (i.e., TMS) were designated as the CTS, while the 15 amino acids N-terminal of the hydrophobic core were designated as the NTS. The borders between the TMS and the CTS and NTS were defined by the intersection of the Hopp-Woods and Kyte-Doolittle definitions of the TMS and are designated the C-border and N-border, respectively.

### Removal of sequence redundancy

We eliminated sequence redundancy within the dataset using BLASTClust with an 80% sequence identity threshold and 80% length coverage [45, 46].

### GO term annotation analysis

The Database for Annotation, Visualization and Integrated Discovery (DAVID) was used to analyze overrepresentation and organize the GO categories [47]. The most representative TAMP-associated cellular functions or compartments with significant enrichment (FDR < 0.001) were selected. Underrepresentation analysis was performed using FuncAssociate [48].

### Statistical enrichment

The enrichment of amino acids at each position was calculated based on the frequency of occurrence when compared to the frequency by which the same amino acid was found in the region outside the TA region for all of the selected proteins. Significant enrichment was defined by calculating the distribution of enrichment ratios across positions for each amino acid. Following, *p*-values were calculated using a parametric approach.

### Enrichment of protein-protein interactions (PPIs)

We downloaded PPIs from BioGRID version 3.4.131 [49], processed the resulting network, and removed self-edges. The enrichment of interactions was calculated using a Monte Carlo approach in which we compared the observed number of interactions involving protein in GO sets with the expected number calculated by randomly selecting proteins from the PPI network. The expected number of interactions was calculated by randomly sampling the same number of proteins in each GO Term and comparing the number of interactions against the number of interactions observed. After normalizing both observed and expected values using the total number of interactions found in each of these two groups, we calculated the logarithm of the fraction observed/expected.

### Conservation analysis

Conserved orthologs were identified as human genes with orthologs in 8 different species (*A*. *thaliana*, *B. taurus*, *C. elegans*, *C. familiaris*, *M. mulatta*, *M. musculus*, *R. norvegicus*, and *S*. *cerevisiae*), as determined by InParanoid [50].

### Plasmid construction

All coding regions of human TAMPs were cloned into pQCXIP, a retroviral expression vector (Clontech), with either the open reading frame (ORF) of EGFP-S65 T or mLumin (kindly provided by Jeanne Hardy, UMass Amherst, USA) upstream of the gene-of-interest. Briefly, both fluorophores were amplified by polymerase chain reaction (PCR) incorporating flanking *Age*I (5′) and *Xho*I (3′) restriction sites. cDNAs of NP_064564 and NP_057648 were synthesized by IDT-DNA in form of gBLOCKS. All other cDNAs (NP_149991, NP_079504, and NP_003797) were purchased from Open Biosystems or Origene. All cDNA were PCR amplified, along with flanking SalI (5′) and BamHI (3′) restriction sites.

cDNAs encoding plant *(A. thaliana)* TAMPs examined in this study were initially obtained from the *A. thaliana* Biological Resource Center (ABRC) (Ohio State University) or RIKEN Bioresource Center and then, using PCR and forward and reverse primers to incorporate the appropriate flanking restriction sites, were sub-cloned downstream of GFP in pRTL2/GFP-MCS. pRTL2/GFP-MCS is a plant expression vector that includes the 35S cauliflower mosaic virus (CMV) promoter and the GFP ORF, followed by a multiple cloning site (MCS) [51]. Modified versions of pRTL2/GFP-TAMP constructs lacking their C-terminal tails were generated by introducing (via PCR site-directed mutagenesis) a stop codon immediately upstream of the tail region. Alternatively, GFP-TAMP constructs consisting of GFP appended to only the C-terminal tail region of plant TAMPs were generated using PCR and the appropriate restriction sites, and then subcloning into pRTL2/GFP-MCS. Plasmids encoding plant TAMPs used for BiFC assays were generated based on the Gateway-compatible vector pDEST-SCYCE/cCFP, which encodes the C-terminal half of CFP [52], and was obtained from ABRC. Complete details on the construction procedures used for generating plasmids encoding any of the various plant TAMPs and modified versions thereof described in this study are available upon request. Other plant expression plasmids used in this study have been described elsewhere, including the following: pRTL2/GFP-AT1G16000 [31], pRTL2/GFP-AT3G63160 [29] and GFP-AT1G55450 [53], encoding GFP fused to the *A. thaliana* TAMPs AT1G16000, AT3G63160, and AT1G55450, respectively; pDEST-VYNE/nVenus-CAT, encoding the nVenus fused to bacterial chloramphenicol acyltransferase, and pDEST-SCYE/cCFP-AT1G16000 and pDEST-SCYE/cCFP-APG1, encoding cCFP fused to AT1G16000 and the chloroplast inner envelope membrane protein albino or pale green mutant 1 (APG1), respectively [29]; pRTL2/BCAT3-mCherry encodes *A. thaliana* branched-chain aminotransferase 3 fused to mCherry [54]; pBIN/ER-RK, encoding mCherry fused to an N-terminal signal sequence and C-terminal ER retrieval signal (−KDEL), and pBIN/VAC-RK, encoding mCherry fused to the vacuolar protein γTIP, and referred to in this study as mCherry-ER and mCherry-Vac, respectively, were both obtained from ABRC (clone no. CD3–959 and CD3–959).

### Mammalian cell lines and culturing

Normal murine mammary gland (NMuMG) cells (generous gift of Jeff Wrana, Lunenfeld-Tanenbaum Research Institute) were cultured in DMEM (Gibco), containing 10 μg/ml bovine insulin (Sigma), 10% fetal bovine serum (FBS) (Gibco), and penicillin/streptomycin (Wisent). The retroviral packaging cell line (Phoenix) was grown in DMEM, supplemented with 10% FBS and penicillin/streptomycin. Cell lines were maintained in a 5% CO_2_ atmosphere at 37 °C. Cells were tested mycoplasma free using a PCR based detection system [55].

### Mammalian cell transfection and transduction

Retrovirus was derived by transient transfection of plasmids encoding putative TAMPs into the Phoenix packaging cell line using TurboFect (ThermoFisher). After 48 h the virus-containing cell medium was filtered (0.45 μm, PALL) and transferred onto NMuMG cells. Stable colonies were selected in 10% FBS/DMEM containing 2 μg/ml puromycin (Sigma).

### Plant material, growth conditions, and transformations

*Nicotiana tabacum* Bright Yellow (BY)-2 suspension-cultured cells were maintained and prepared for (co) transformation via biolistic particle bombardment using a Bio-Rad PDS system 1000/HE, as described previously [56]. Transient (co) transformations were performed using 1–2 μg of plasmid DNA. Following bombardment, cells were incubated for 4–6 h to allow for expression and sorting of the introduced gene product(s) and then processed for (immuno) fluorescence CLSM. *N. benthamiana* plants were grown in soil, and leaves of 28-day-old tobacco plants were infiltrated with *Agrobacterium tumefaciens* (strain LBA4404) carrying a selected binary plasmid, followed (~ 3 days later) by CLSM imaging.

### Microscopy

NMuMG cells expressing putative TAMPs were seeded in 384-well microplates (CellCarrier-384 ultra, B128 SRI/160, Perkin Elmer), and allowed to grow for 24 h prior to staining with both nuclear dye DRAQ5™ (Biostatus) and Mitotracker Red or Mitotracker Green (ThermoFisher) according to the manufacturer’s instructions. Plates were imaged using PerkinElmer OPERA® QHS spinning-disk automated confocal microscope with 40× water objective.

Plant BY-2 cells and tobacco leaves were processed for CLSM imaging, including immunostaining with mitochondrial E1β antibodies (provided by T. Elthon, University of Nebraska-Lincoln) and ER staining with ConA (conjugated to Alexa 594 [Molecular Probes], as previously described [57]. Imaging of plant cells was carried out using a Leica DM RBE microscope equipped with a 63x Plan Apochromat oil-immersion objective and TCS SP2 scanning head (Leica Microsystems). Excitations and emission signals for fluorescent proteins and/or chlorophyll autofluorescence collected sequentially as single optical sections are the same as those described in Gidda et al. [57]; single-labeling experiments showed no detectable crossover at the settings used for data collection. Micrographs shown in figures are representative of the results obtained from analyzing ≥25 independently transformed cells/cell areas from at least three separate experiments.

### Carbonate extraction and western blot analysis

Isolation of subcellular membranes by means of sodium carbonate treatment was carried out as previously described [13]. Cells were harvested in a hypotonic buffer (20 mM HEPES, 2 mM EDTA, 2 mM MgCl_2_, 10 mM KCl, 1 mM DTT,1 mM PMSF, protease inhibitor (Sigma): chymostatin (C7268), antipain (A6191), leupeptin (L2884), pepstatin A (P5318), aprotinin (A4529)), broke up by a Dounce homogenizer (Wheaton) (20 strokes) and centrifuged (3000 rpm, 10 min, 4 °C, Eppendorf 5424R). Subsequently, the supernatant was centrifuged (40,000 rpm, 60 min, at 4 °C, TLA100, Optima MAX-XP, Beckman Coulter) to separate the cytosol from the membranes. The remaining pellet was incubated with pre-chilled 0.1 M sodium carbonate buffer (pH 11.5) (BioShop) 60 min at 4 °C on an end over end shaker and centrifuged (52,000 rpm, 10 min, at 4 °C, TLA100, Optima MAX-XP, Beckman Coulter) to separate peripheral proteins from integral membranes proteins. Cell equivalents were loaded on a 10% SDS-PAGE and transferred onto a nitrocellulose membrane (0.45 μm, GE Healthcare, Amersham) using BIO-RAD Trans-Blot Turbo Transfer System (25 V, 1.0A, 30 min). Antibodies for detection were GFP (4B10, Cell signalling, 1:1000), RFP (RF5R, ThermoFisher, 1:2500), Hsp60 (Mouse, in house, 1:2000), and GRP78 (SPA-826, polyclonal, StressGen Biotechnologies Corp., 1:1000) followed by HRP-conjugated donkey anti-mouse or donkey anti-rabbit secondary antibody (715–035-151 and 711–035-152, respectively, Jackson ImmunoResearch, 1:10,000). Antibody dilutions were used as recommended by their manufacturers in 5% BSA (BioShop). Western Blot was developed by enhanced chemiluminescence (EZ-ECL, BI Biological Industries) using MicroChemi 4.2 imager (DNR Bio Imaging Systems). Controls for fraction separation (Additional file 11: Figure S7). Data are representative of three sodium carbonate extractions.

### Bimolecular fluorescence complementation (BiFC) assay

BiFC assays for assessing putative plant TAMP topology in tobacco leaf cells were performed as previously described [29]. Briefly, leaves were infiltrated with *Agrobacterium*-containing plasmids encoding nVenus-CAT and a cCFP-TAMP fusion protein, as well as either Cherry-Mito, Cherry-ER or Cherry-Vac, serving as an organelle marker for mitochondria, ER or vacuoles, respectively. Transformed cells in leaf areas were visualized by CLSM and both reconstituted BiFC (nVenus/cCFP) and endogenous chlorophyll or Cherry fluorescence signals were collected with identical image acquisition settings for all samples analyzed. CLSM acquisition settings, amounts of *Agrobacterium* infiltrated, and post-infiltration times were chosen based preliminary optimization experiments aimed at minimizing the possibility of non-specific interactions based on guidelines described by Stefano et al. [25]. None of the BiFC constructs examined in this study resulted in a BiFC signal when expressed alone, as expected.

## Supplementary information

**Additional file 1: Table S1.** List of all proteins used for training and test set.

**Additional file 2: Figure S1.** Comparison of the relative utility of different hydrophobicity scales to establish a classifier to identify tail-anchored proteins in the human genome. The height of the bars indicates the percentage of the indicated protein type classified as a putative TAMP by the indicated hydrophobicity scales. The optimal scale passes the largest percentage of TAMPs and the smallest percentage of other proteins.

**Additional file 3: Table S2.** TAMPfinder datasets for H. sapiens, A. thaliana, and S. cerevisiae.

**Additional file 4: Table S3.** Direct comparison of Kalbfleisch and TAMPfinder for potential tail-anchored proteins in H. sapiens using UniProt data of single-pass IV membrane proteins.

**Additional file 5: Figure S2.** Gene Ontology annotations of predicted TAMPs. (a) Fraction of proteins with the selected Gene Ontology annotations related to cell compartments. Even though TAMPfinder identified many more putative TAMPs the distribution of GO annotations was similar for putative TAMPs identified using TAMPfinder or by Kalbfleisch et al. (b) Comparison of GO terms associated with putative TAMPs identified using TAMPfinder program in comparison with those found previously by Kalbfleisch et al.. Both exclusive and common proteins were analyzed in terms of GO.

**Additional file 6: Figure S3.** Putative A. thaliana TAMPs are enriched in GO terms associated with membranes. Only significantly enriched compartments (FDR < 0.001) are considered. Colors indicate GO terms with similar protein membership. The number of predicted TAMPs for each annotation is indicated to the right of the bar.

**Additional file 7: Figure S4.** Differences in amino acids enrichment at specific positions of the TA sequences between H. sapiens and A. thaliana using N- and C-border alignments. Red squares show an overrepresentation of amino acids in the H. sapiens dataset at specific positions compared to A. thaliana, while light red squares display enrichment of amino acids at specific positions in the A. thaliana dataset. Dark red squares show no significant difference between species (both). Similarly, underrepresentation of amino acids in the both datasets at specific positions was displayed (shade of blue). Significant enrichment was defined by calculating z-score of enrichment ratios (H. sapiens/A. thaliana) across positions for each amino acid. Cut-off was defined as *P* < 0.002.

**Additional file 8: Figure S5.** Statistical significance at each positions of the TA sequences between H. sapiens and A. thaliana using N- and C-border alignments. To estimate the significance of the enrichment of amino acids at the different positions in TA region, the sequences of the TA regions were aligned on either the N- or C-border as indicated at the right and the enrichment from Fig. 5 of occurrence for all 20 amino acids at the indicated locations were defined by calculating the distribution of enrichment ratios across positions for each amino acid. Following, *p*-values were calculated using a parametric approach. Red indicates overrepresentation; blue indicates underrepresentation at *P* < 0.05. Amino acid identities are indicated in single letter code at the right of the panels.

**Additional file 9: Figure S6.** Profile of conservation across different human protein lengths. Each cell shows the percentage of orthologs (a proxy of conservation) from a given species (row) among proteins with a specific length (column). Shown are conservation profiles of proteins localized in (A) cytoplasm and (B) membranes.

**Additional file 10: Table S4.** Performance of TAMPfinder program in comparison with Kalbfleisch et al. Both exclusive and common membership was analyzed in terms of GO annotation.

**Additional file 11: Figure S7.** Sodium carbonate extraction of mitochondrial matrix proteins used as controls. Sodium carbonate extraction of the indicated control proteins demonstrates that the extraction procedure released matrix proteins from mitochondria. Total (T) cell lysate prepared from NMuMG cells was fractionated into a supernatant fraction (S) containing cytosolic proteins and the proteins inside mitochondria were pelleted and then separated into fractions containing peripheral and luminal proteins.

## Data Availability

The complete annotated sequences for all of the identified TA regions are included as Additional file 3: Table S2. In addition, all three datasets as well as the most current ones are available as web versions at [43].

## Abbreviations

BiFC: bimolecular fluorescence complementation
BioGRID: Biological General Repository for Interaction Datasets
CTS: carboxyl-terminal sequence
FDR: False discovery rate
GO: Gene ontology
NTS: amino (NH_2_)-terminal sequence
SUBA: subcellular localisation database for Arabidopsis proteins
TAMP: tail-anchored membrane proteins
TMS: transmembrane sequence
VerSeDa: Vertebrate Secretome database
