# Supplementary figures and images for: Genome-wide analysis of Homo sapiens, Arabidopsis thaliana, and Saccharomyces cerevisiae reveals novel attributes of tail-anchored membrane proteins
Source: BMC Genomics. 2019 Nov 11;20:835. doi: 10.1186/s12864-019-6232-x (PMC6849228; doi:10.1186/s12864-019-6232-x)

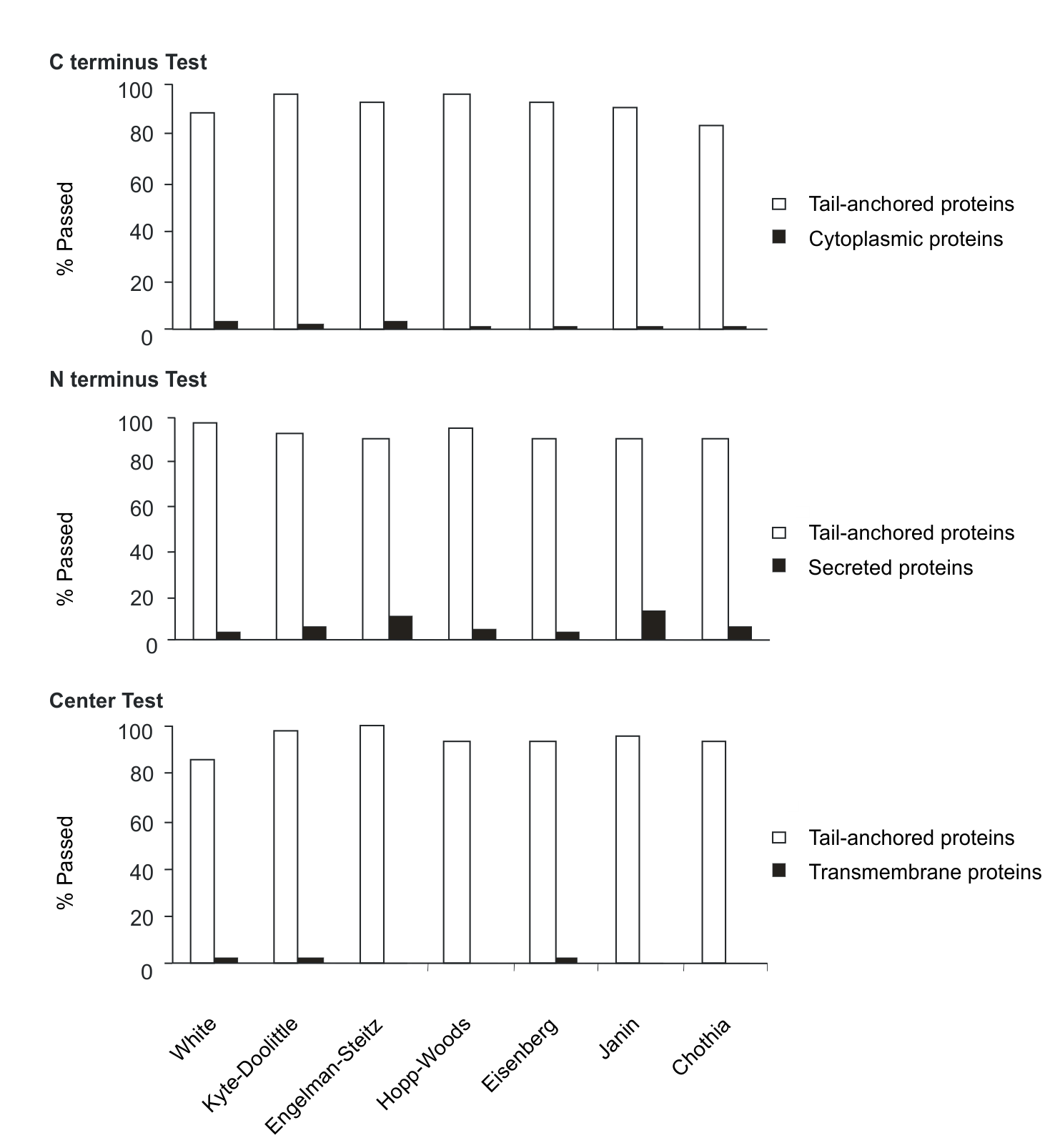

Supplement: Supplementary file 2 — Additional file 2: Figure S1. Comparison of the relative utility of different hydrophobicity scales to establish a classifier to identify tail-anchored proteins in the human genome. The height of the bars indicates the percentage of the indicated protein type classified as a putative TAMP by the indicated hydrophobicity scales. The optimal scale passes the largest percentage of TAMPs and the smallest percentage of other proteins. [file 12864_2019_6232_MOESM2_ESM.tif]

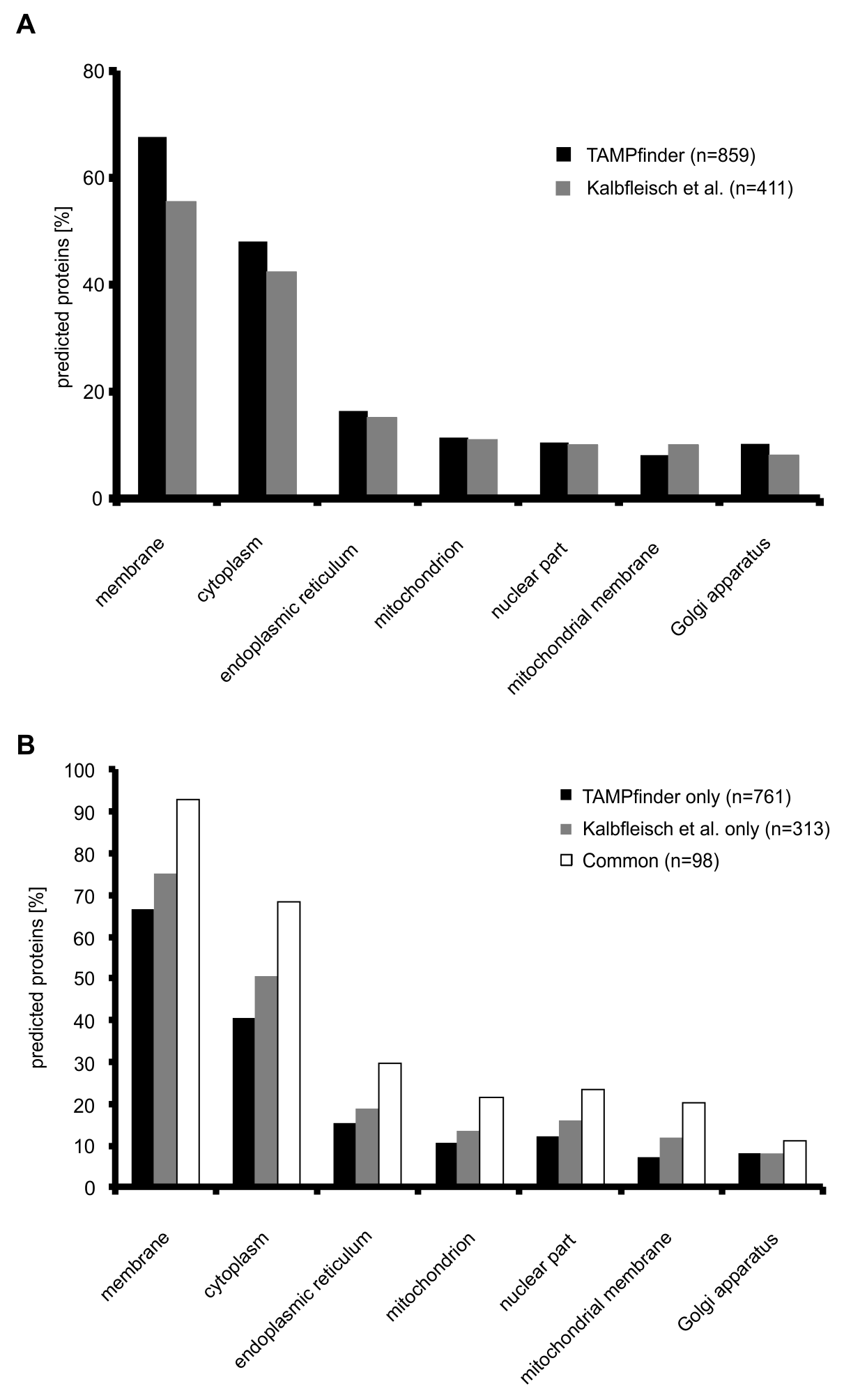

Supplement: Supplementary file 5 — Additional file 5: Figure S2. Gene Ontology annotations of predicted TAMPs. (a) Fraction of proteins with the selected Gene Ontology annotations related to cell compartments. Even though TAMPfinder identified many more putative TAMPs the distribution of GO annotations was similar for putative TAMPs identified using TAMPfinder or by Kalbfleisch et al. (b) Comparison of GO terms associated with putative TAMPs identified using TAMPfinder program in comparison with those found previously by Kalbfleisch et al.. Both exclusive and common proteins were analyzed in terms of GO. [file 12864_2019_6232_MOESM5_ESM.tif]

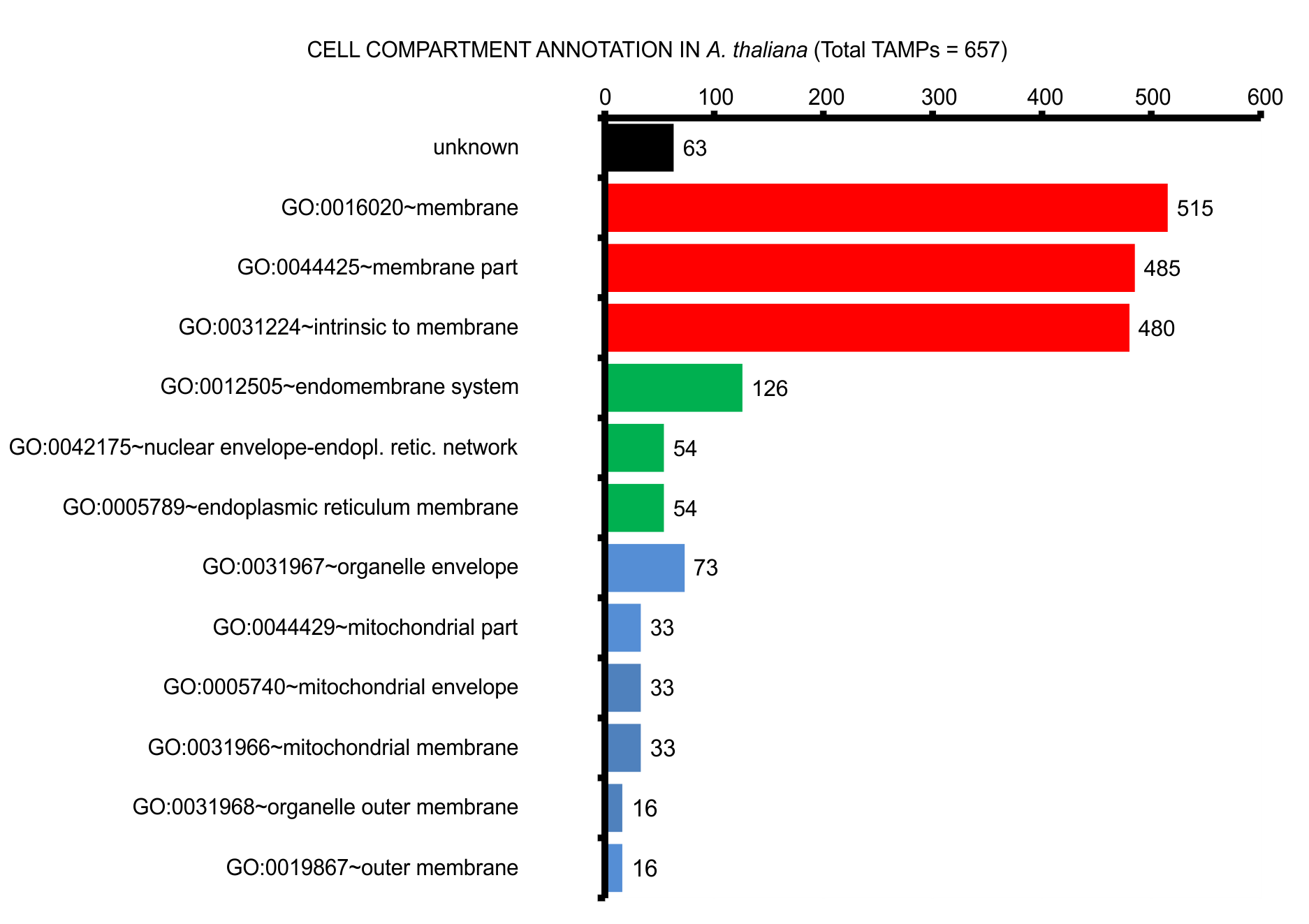

Supplement: Supplementary file 6 — Additional file 6: Figure S3. Putative A. thaliana TAMPs are enriched in GO terms associated with membranes. Only significantly enriched compartments (FDR < 0.001) are considered. Colors indicate GO terms with similar protein membership. The number of predicted TAMPs for each annotation is indicated to the right of the bar. [file 12864_2019_6232_MOESM6_ESM.tif]

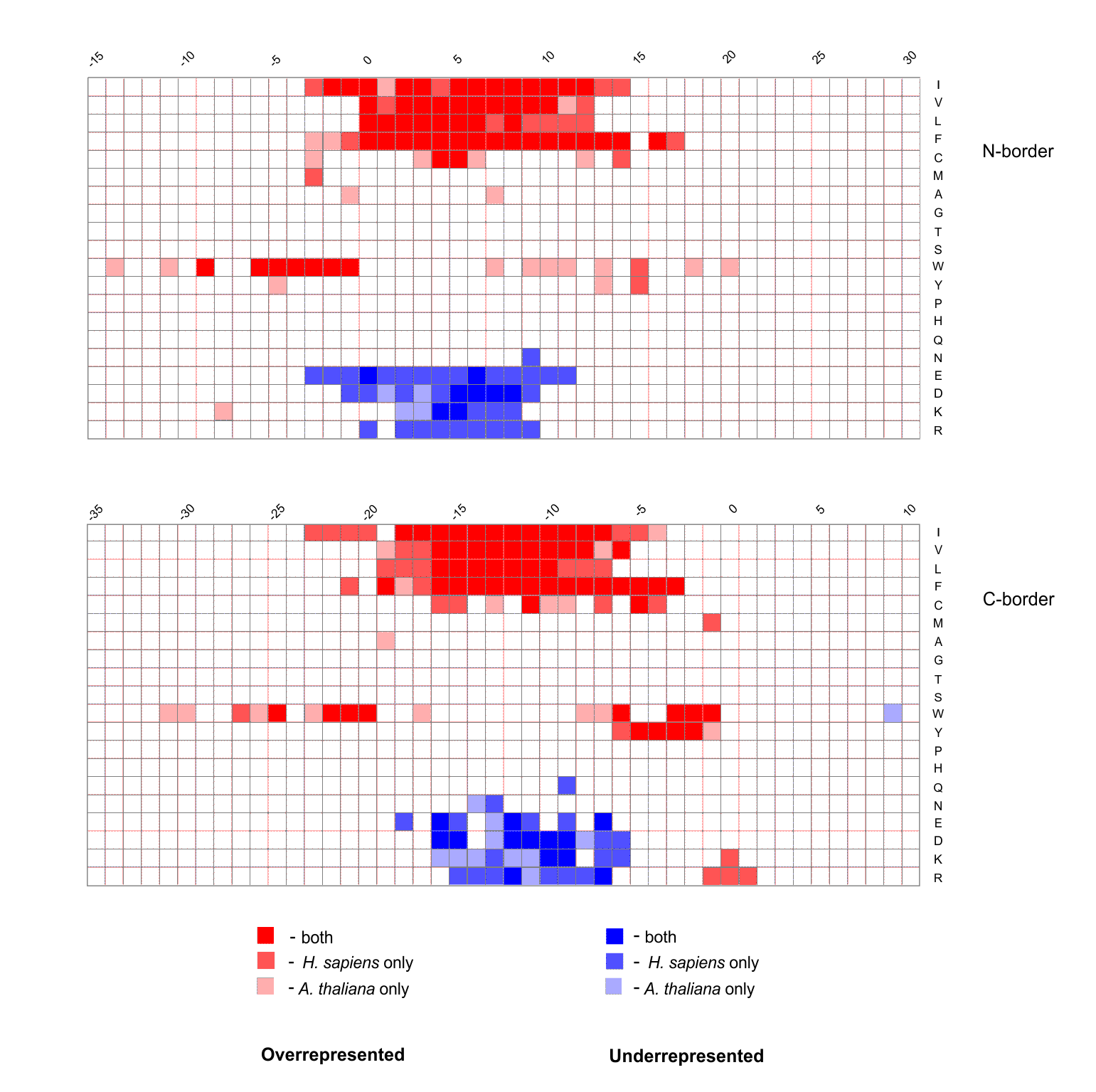

Supplement: Supplementary file 7 — Additional file 7: Figure S4. Differences in amino acids enrichment at specific positions of the TA sequences between H. sapiens and A. thaliana using N- and C-border alignments. Red squares show an overrepresentation of amino acids in the H. sapiens dataset at specific positions compared to A. thaliana, while light red squares display enrichment of amino acids at specific positions in the A. thaliana dataset. Dark red squares show no significant difference between species (both). Similarly, underrepresentation of amino acids in the both datasets at specific positions was displayed (shade of blue). Significant enrichment was defined by calculating z-score of enrichment ratios (H. sapiens/A. thaliana) across positions for each amino acid. Cut-off was defined as P < 0.002. [file 12864_2019_6232_MOESM7_ESM.tif]

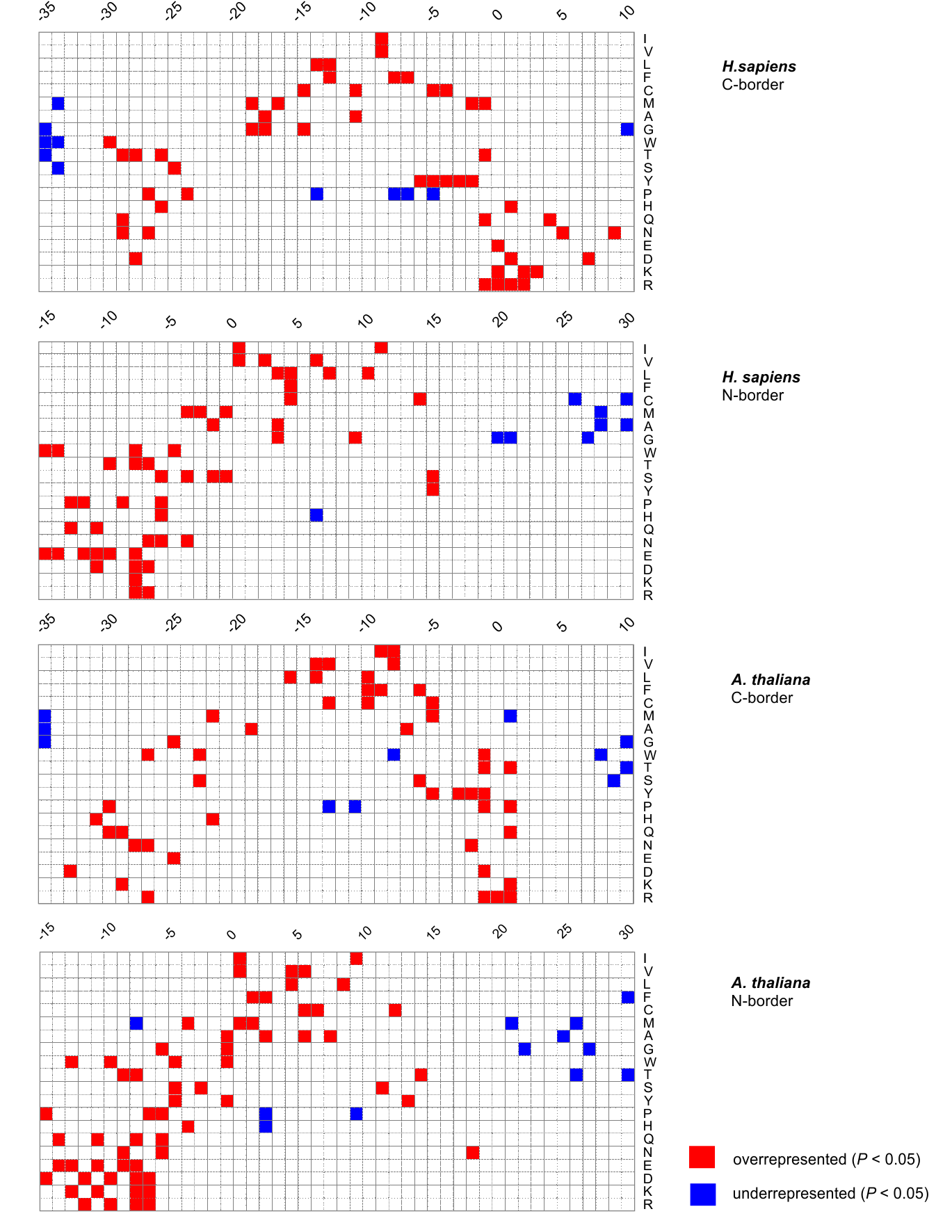

Supplement: Supplementary file 8 — Additional file 8: Figure S5. Statistical significance at each positions of the TA sequences between H. sapiens and A. thaliana using N- and C-border alignments. To estimate the significance of the enrichment of amino acids at the different positions in TA region, the sequences of the TA regions were aligned on either the N- or C-border as indicated at the right and the enrichment from Fig. 5 of occurrence for all 20 amino acids at the indicated locations were defined by calculating the distribution of enrichment ratios across positions for each amino acid. Following, p-values were calculated using a parametric approach. Red indicates overrepresentation; blue indicates underrepresentation at P < 0.05. Amino acid identities are indicated in single letter code at the right of the panels. [file 12864_2019_6232_MOESM8_ESM.tif]

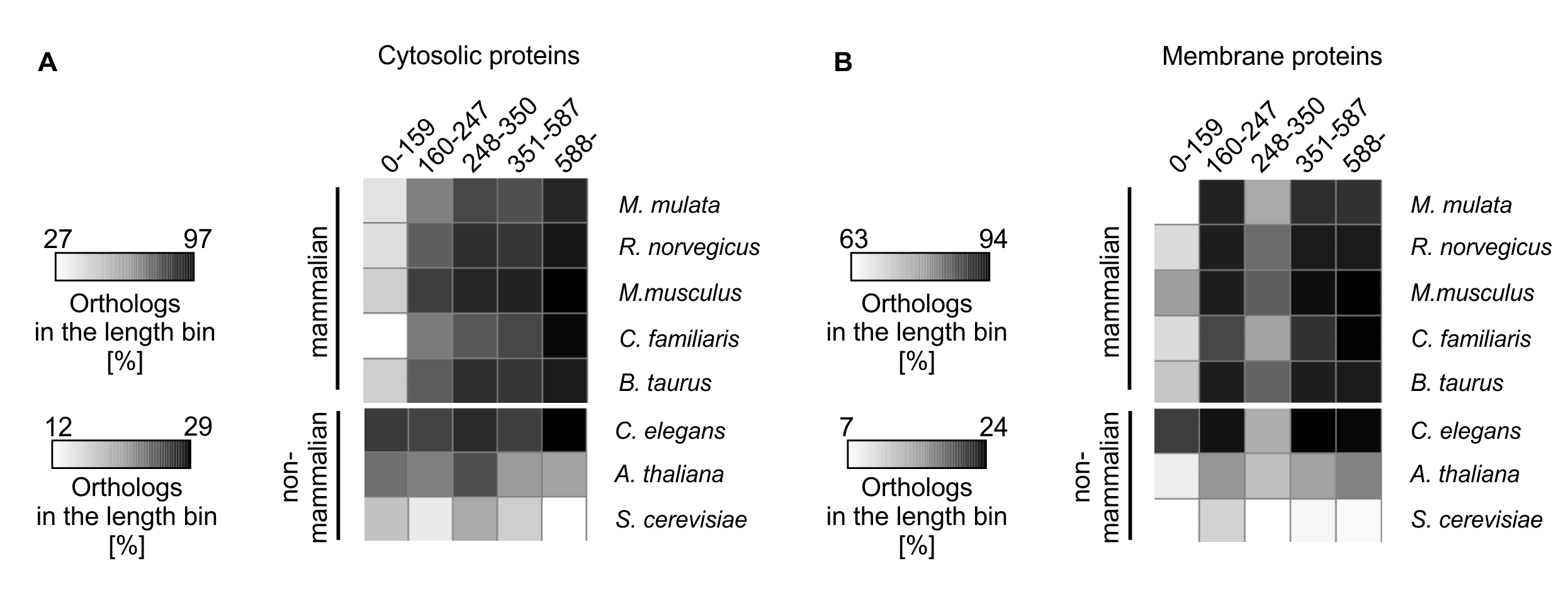

Supplement: Supplementary file 9 — Additional file 9: Figure S6. Profile of conservation across different human protein lengths. Each cell shows the percentage of orthologs (a proxy of conservation) from a given species (row) among proteins with a specific length (column). Shown are conservation profiles of proteins localized in (A) cytoplasm and (B) membranes. [file 12864_2019_6232_MOESM9_ESM.tif]

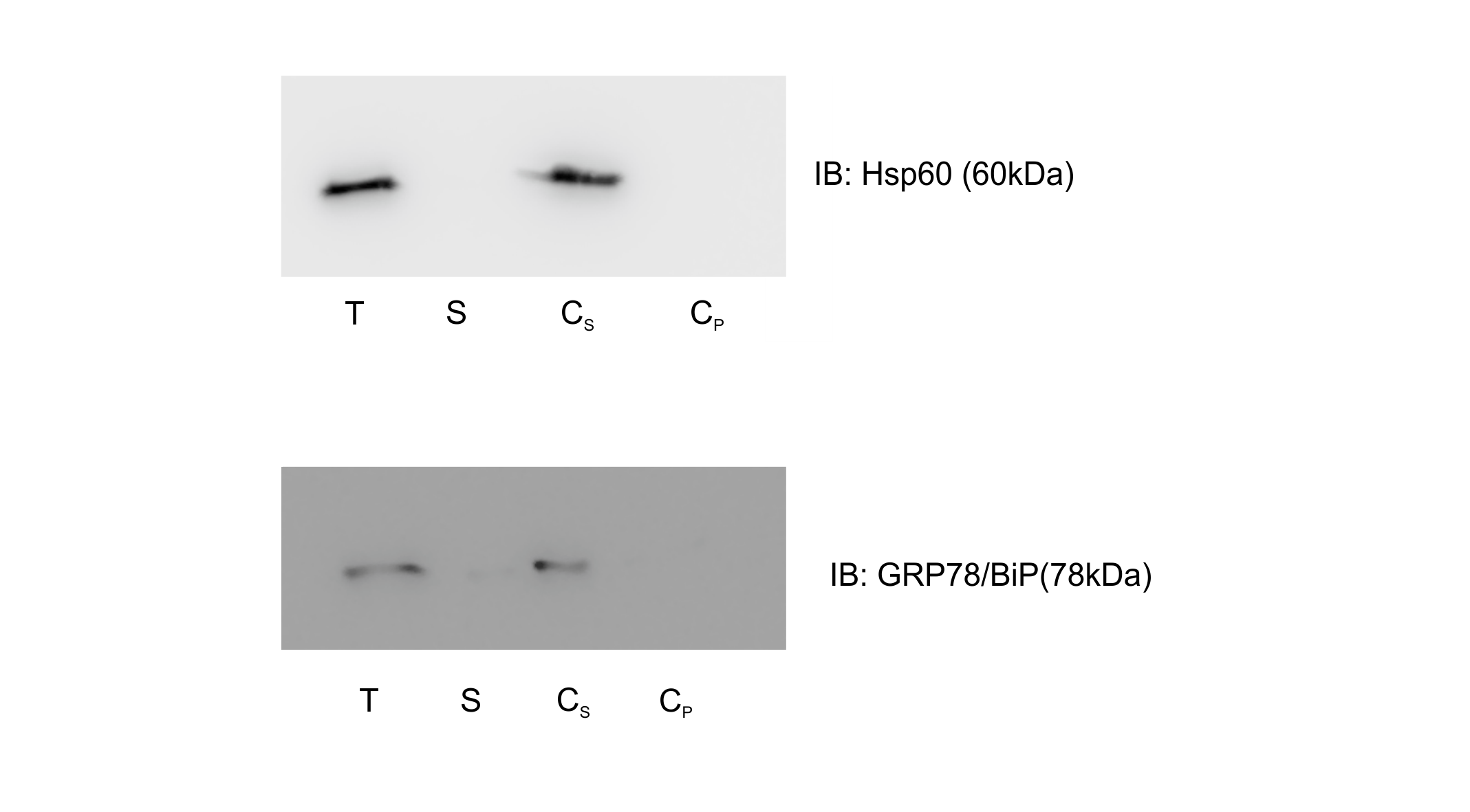

Supplement: Supplementary file 11 — Additional file 11: Figure S7. Sodium carbonate extraction of mitochondrial matrix proteins used as controls. Sodium carbonate extraction of the indicated control proteins demonstrates that the extraction procedure released matrix proteins from mitochondria. Total (T) cell lysate prepared from NMuMG cells was fractionated into a supernatant fraction (S) containing cytosolic proteins and the proteins inside mitochondria were pelleted and then separated into fractions containing peripheral and luminal proteins. [file 12864_2019_6232_MOESM11_ESM.tif]
